# Synergistic Effects of Solid Electrolyte Mild Sintering and Lithium Surface Passivation for Enhanced Lithium Metal Cycling in All‐Solid‐State Batteries

**DOI:** 10.1002/advs.202521791

**Published:** 2026-01-08

**Authors:** Jinsong Zhang, Robin N. Wullich, Thomas J. Schmidt, Mario El Kazzi

**Affiliations:** ^1^ PSI Center for Energy and Environmental Sciences, Paul Scherrer Institute Villigen CH‐5232 Switzerland; ^2^ Institute For Molecular Physical Science ETH Zurich Zurich CH‐8093 Switzerland

**Keywords:** all‐solid‐state batteries, cycling performance, Li_6_PS_5_Cl, lithium metal, sintering treatment, surface passivation

## Abstract

The argyrodite‐type solid electrolyte (SE) Li_6_PS_5_Cl (LPSCl), recognized for its high ionic conductivity and low‐temperature processability, offers substantial potential for enabling lithium metal anodes in all‐solid‐state batteries (ASSBs), promising high energy densities with enhanced safety. However, lithium dendrite penetration and unstable solid electrolyte interphase (SEI) formation hinder stable cycling at high current densities. This work presents a synergistic strategy to address these challenges by combining mild sintering of LPSCl pellets with the deposition of a lithium fluoride (LiF) passivation layer on 50 µm thick lithium metal. Optimized sintering at 80°C improves surface uniformity and densifies the LPSCl pellets, reducing porosity and increasing ionic conductivity. Complementarily, the deposition of a uniform 65 nm LiF layer on lithium via electron beam evaporation, reduces interfacial resistance, and stabilizes SEI formation. This dual modification doubles the critical current density of lithium symmetric cells from 1.1 to 2.2 mA cm^−2^. In full cells configurations with LiNi_0.8_Co_0.1_Mn_0.1_O_2_ (NCM811) cathodes, remarkable cycling stability is achieved over 2700 cycles (at 1 mA cm^−2^, 1.5 mAh cm^−2^), with 75% capacity retained after 1500 cycles. This study provides a practical approach for improving both SE pellet quality and lithium‐SE interfacial stability, paving the way for the reliable implementation of thin lithium metal in next‐generation ASSBs.

## Graphical abstract

This study proposes a synergistic strategy combining mild sintering of solid electrolyte pellets with lithium metal surface passivation, effectively suppressing dendrite growth, and enhancing interfacial stability. This approach doubles the critical current density of symmetric cells (1.1 to 2.2 mA cm^−2^) and extends full cells cycle life at 1 mA cm^−2^ from 16 to over 2700 cycles.

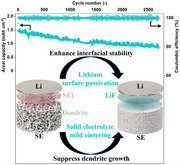

## 1 Introduction

The evolution of lithium‐based energy storage technologies has been pivotal in advancing portable electronics, electric vehicles, and grid‐scale storage [1]. Conventional lithium‐ion batteries, which typically employ liquid organic electrolytes and graphite anodes, have become the dominant technology due to their long cycle life and reliability [2]. However, their reliance on flammable liquid electrolytes and limitations in energy density pose significant challenges in meeting growing demands for safety and performance [3, 4].

In response to these limitations, all‐solid‐state batteries (ASSBs) have emerged as a promising next‐generation technology. By replacing flammable liquid electrolytes with non‐flammable solid electrolytes (SE) and incorporating a thin lithium metal anodes, ASSBs offer the dual advantages of enhanced intrinsic safety and significantly higher energy densities (> 500 Wh kg^−1^) [5, 6]. Among the various SE candidates, argyrodite‐type Li_6_PS_5_Cl (LPSCl) has garnered considerable attention due to its high ionic conductivity surpassing 1 mS cm^−1^ and facile processability at room temperature [7].

Despite these advantages, lithium metal ASSBs face critical challenges. The inhomogeneous plating and stripping of lithium often result in dendrite formation, undermining cycling stability [8]. Although solid electrolytes are expected to suppress dendrites growth owing to their high mechanical stiffness, practical limitations arise from SE separator microstructure and interfacial chemical incompatibility. For instance, pore‐percolation zone formed during pellet fabrication [9], together with plating‐induced cracks and spallation, provide pathways for lithium dendrites penetration and short‐circuits [10, 11, 12]. Furthermore, the (electro‐)chemical instability of the Li|LPSCl interface exacerbates performance degradation. The narrow electrochemical stability window of LPSCl promotes decomposition into Li_2_S, LiCl, and Li_3_P, leading to the formation of a non‐beneficial solid electrolyte interphase (SEI) [13, 14, 15]. Continuous SEI growth significantly increases interfacial resistance, obstructs Li‐ion transport, and ultimately accelerate performance decay [16].

To address these issues, strategies have focused on chemically modifying SEs or interfaces and densifying SE pellets. Doping LPSCl with metals such as copper and magnesium has been shown to improve ionic conductivity and hinder the redox reactions with lithium [17, 18]. Functional additives have also been introduced to stabilize the SEI and mitigates dendrite growth [19, 20]. For example, Zhang et al. combined LPSCl with 7 wt.% polycaprolactone‐based binder at 80°C, which is ionically conductive yet electronically insulative, to fabricate a compact SE membranes with enhanced interfacial stability [21]. Coskun et al. employed 2.5 wt.% pre‐lithiated trithiocyanuric acid (Li_3_TCA) organic additive into the LPSCl to reduce Li_2_S accumulation at the interface [22].

Alternatively, tailoring SE microstructure, without altering the chemical composition, has proven effective in enhancing the critical current density (CCD). Mitlin et al. demonstrated that wet ball milling of LPSCl powders refines pore and grain size distribution, improving pellet uniformity and interfacial contact [23]. Yao et al. further showed that high temperature sintering enhances LPSCl pellet density and surface flatness [24]. However, LPSCl sintering conducted at 550°C risks amorphization, phase transformations, decomposition, and H_2_S release [25, 26, 27, 28]. To overcome these drawbacks, Bruce et al. employed short spark plasma sintering (5 min at 400°C), achieving pellet density > 99% and plating CCD of 9 mA cm^−2^ in symmetric cell [29]. Nevertheless, translating such advances into robust full‐cell performance remains scarce, particularly with respect to achieving long cycle lifetime while maintaining high stability at elevated current densities.

Given the critical role of the SE pellet density and microstructure, it is important to explore mild sintering under low applied pressures to densify LPSCl without inducing structural changes or decomposition [30, 31]. At the same time, stabilizing the Li|SE interface remains imperative by employing barrier / lithiophobic layers. For instance, recent studies have focused on artificial SEI containing lithium fluoride (LiF), which help mitigate interfacial side reactions by blocking electron leakage and inhibit lithium dendrite growth due to its high interfacial energy with lithium (73.28 meV Å^−2^). Reported approaches include LiF‐coated core‐shell SE particles and a melt‐infusion method that introduces lithium trifluorosulfonylimide (LiTFSI) onto LPSCl particles, however, these strategies can compromise the ionic conductivity of SE [32, 33]. LiF coatings prepared via wet‐chemical or dry thin‐film deposition processes have been reported to inhibit lithium filament under high current densities in quasi‐solid and solid electrolytes, including garnet‐based and sulfide‐based systems [34, 35, 36, 37]. However, when paired with LPSCl, LiF deposited by evaporation has surprisingly shown limited success. Jung et al. reported that the LiF passivation layers suffered from mechanical damages under stack pressure, leading to non‐uniform contact and aggravated side reactions [38]. These observations indicate that the effectiveness of LiF strongly depends on the underlying SE pellet quality and the integrity of the interlayer, and simultaneously underscore the need for approaches that preserve the intrinsic properties of the solid electrolyte.

In this work, we present a combined approach and a comprehensive study to enhancing lithium metal cycling in ASSBs through mild sintering of LPSCl pellets with thin LiF surface passivation. Sintering treatments at controlled temperatures and pressures are used to optimize pellet density and surface morphology, while LiF coatings with varied thicknesses and coverage are deposited on 50 µm lithium foils to investigate their effect on interfacial contact and SEI evolution. Finally, we demonstrate that the synergy between optimized LPSCl sintering and LiF passivation enables superior cycling stability in both lithium symmetric cells and full cells employing LiNi_0.8_Co_0.1_Mn_0.1_O_2_ (NCM811) as cathodes, achieving (i) over 2700 cycles at 1 mA cm^−2^ and 1.5 mAh cm^−2^ with 75% capacity retention after 1500 cycles, and (ii) more than 450 cycles at 1.5 mA cm^−2^ and 1.5 mAh cm^−2^.

## 2 Results and Discussion

**FIGURE 1:**
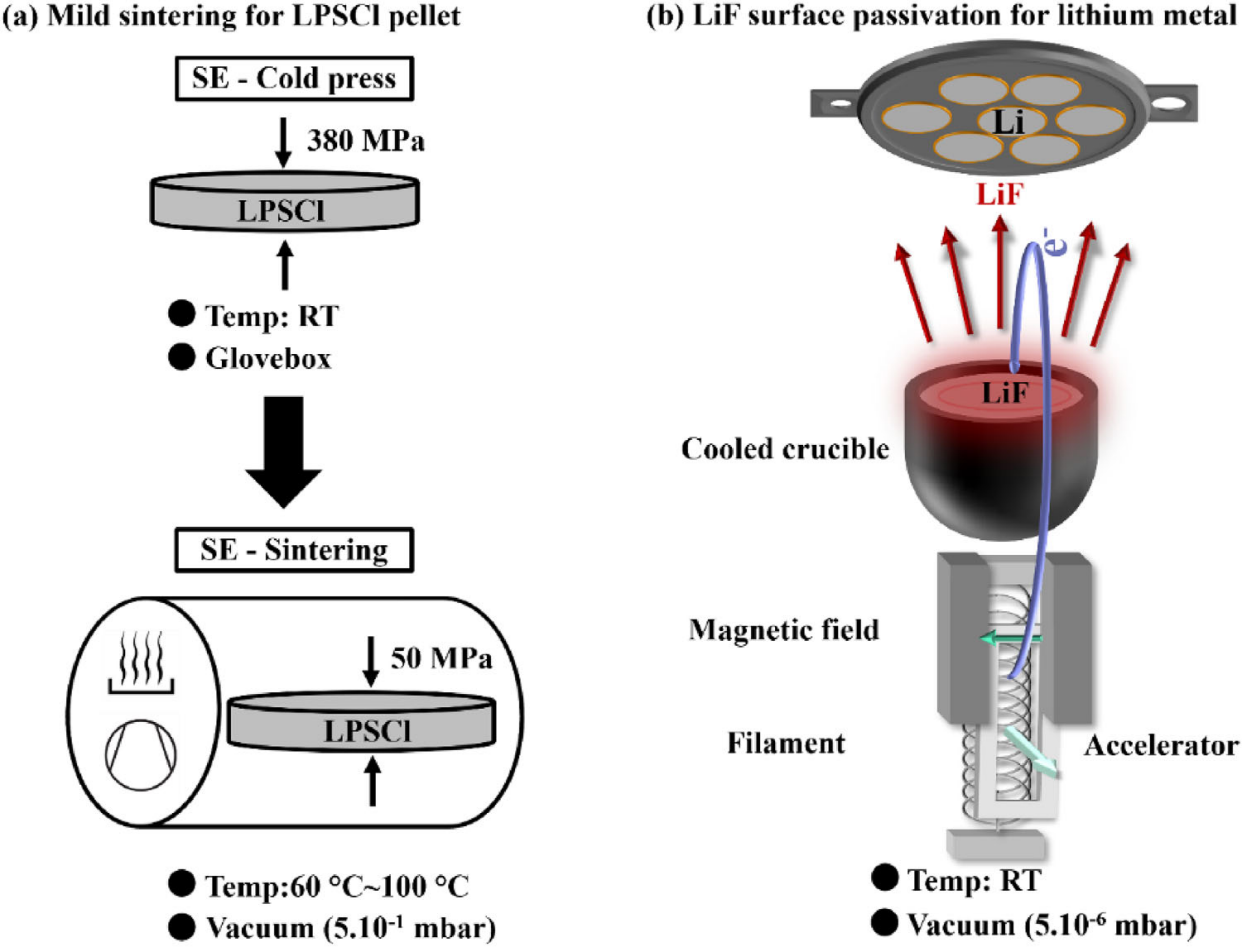
Schematic illustration of (a) the LPSCl pellets manufacturing and the mild sintering workflow, (b) LiF surface passivation of lithium metal by electron‐beam evaporation under 5.10^−6^ mbar at room temperature.

Figure 1 illustrates the preparation procedures for the solid electrolyte (SE) pellets and the LiF surface passivation of the 50 µm thick lithium metal used in this study. (a) Two types of SE pellets were fabricated: (i) cold‐pressed pellets, obtained by uniaxially pressing Li_6_PS_5_Cl (LPSCl) powder at 380 MPa at room temperature inside the glovebox; and (ii) sintered pellets, produced by transferring the cold‐pressed SE into a vacuum chamber (5.10^−1^ mbar) connected to the glovebox and subsequently pressing at 50 MPa for 6 h under different mild temperatures (60°C, 80°C, and 100°C). These are hereafter referred to as 60°C sintered, 80°C sintered, and 100°C sintered LPSCl, respectively. (b) Schematic of the electron‐beam evaporation setup employed to deposit thin lithium fluoride (LiF) layers (under 5.10^−6^ mbar) onto the surface of lithium metal at room temperature, forming homogeneous LiF passivation layers.

### 2.1 Mild‐Sintering Treatment

**FIGURE 2:**
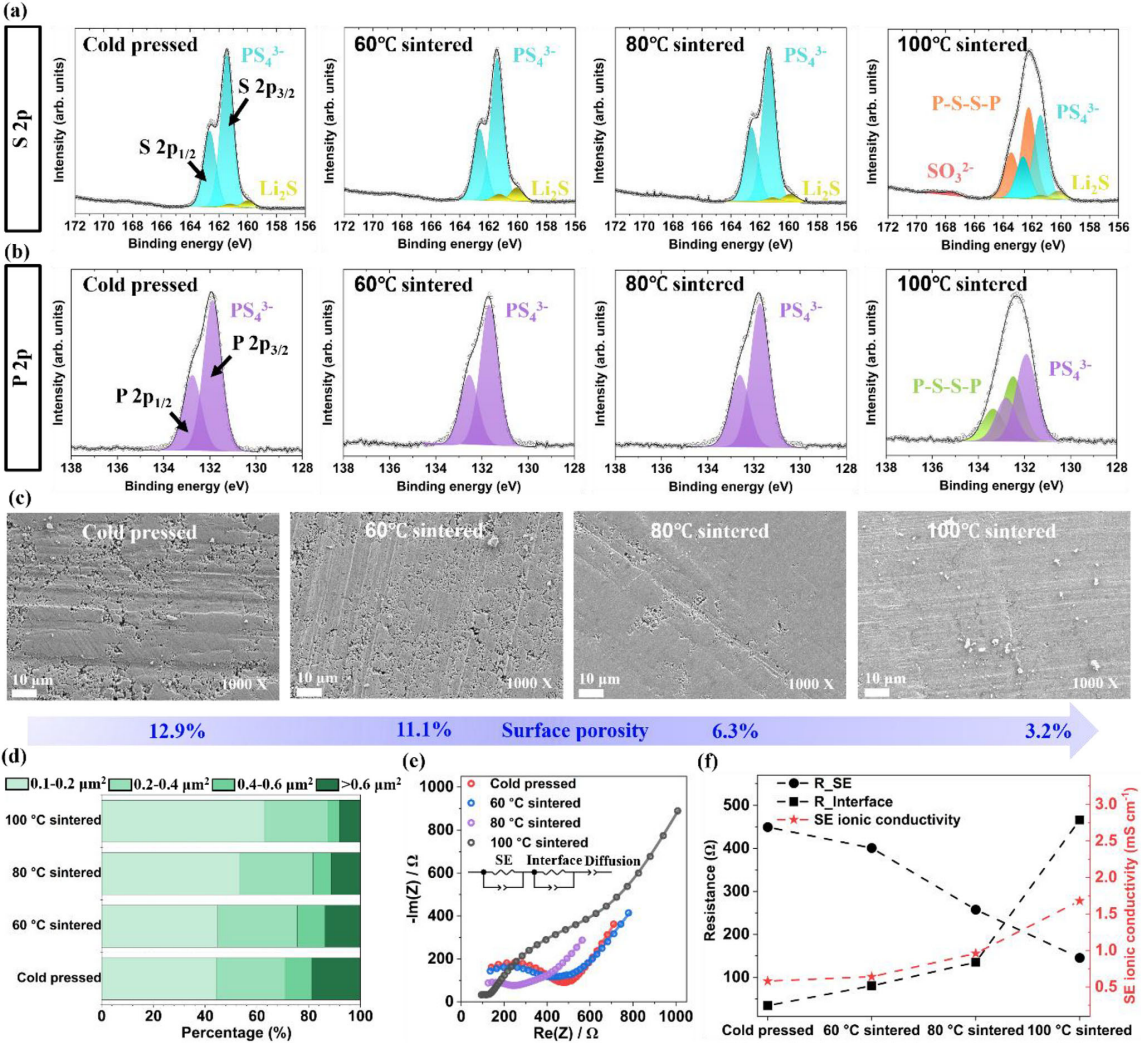
Comparison of cold‐pressed and post‐sintered LPSCl pellets (sintered at 60°C, 80°C, and 100°C). XPS spectra of (a) S 2p, (b) P 2p, (c) SEM images of LPSCl pellet surface morphology, (d) Pore size distribution on LPSCl pellets surface, (e) Nyquist plots and fits of EIS spectra, (f) SE and interface resistance, along with the ionic conductivity of LPSCl SE.

The X‐ray diffraction (XRD) patterns of cold‐pressed and sintered LPSCl pellets presented in Figure S1 showed no changes, peak shifts, or the appearance of new reflection peaks, confirming the structural stability of LPSCl after mild sintering treatments up to 100°C. A LiCl impurity was already detected in the cold‐pressed pellets and remained unchanged after sintering. To further probe the surface chemistry, X‐ray photoelectron spectroscopy (XPS) analysis was conducted to examine the chemical composition of the LPSCl pellets and its evolution after sintering. The most notable changes were observed in S 2p and P 2p spectra (Figure 2a,b). For cold‐pressed LPSCl, the fitted S 2p spectra shows the presence of two S 2p_3/2_ peaks, a dominant one at 161.5 eV corresponding to PS_4_^3−^ units in LPSCl, and a minor peak at 160 eV attributed to Li_2_S. The fitted P 2p spectra exhibited rather a single P 2p_3/2_ peak at 131.9 eV also assigned to PS_4_^3−^. Following the sintering under 60°C and 80°C, both spectra remained largely unchanged. In contrast, the 100°C sintered LPSCl pellets displayed distinct modifications, including additional S 2p_3/2_ peaks corresponding to bridging sulfur (P‐S‐S‐P) at 162.3 eV, and SO_3_^2−^ species at 167.6 eV. Correspondingly, the P 2p spectra revealed new P 2p_3/2_ peak at 132.5 eV, also assigned to P‐S‐S‐P species, consistent with the reported binding energies [13]. To elucidate the formation mechanism, the Li 1s, O 1s, C 1s, and Cl 2p spectra were analyzed, as shown in Figures S2–S5. The full width at half maximum (FWHM) of the Li 1s peak increased from 1.58 to 1.76 after sintering at 100°C. Meanwhile, the binding energy difference between the O 1s and Li 1s peaks was 476.4 eV, matching the characteristic value of LiOH (476.37 ±  0.1 eV) reported by Wood et al. [39]. No significant changes were observed in the C 1s and Cl 2p spectra. Therefore, these spectra changes indicate the formation of LiOH, leading to lithium depletion in LPSCl and subsequent generation of bridging sulfur (P‐S‐S‐P) species, caused by exposure to an oxidative and moist environment [25, 40, 41]. Simultaneously, the same environment induced surface oxidation of LPSCl to produce SO_3_^2−^ species. The source of moisture was linked to the hygroscopic PEEK insulating die [42], used during the LPSCl pellets manufacturing and sintering. At the elevated temperature above 100°C, absorbed moisture is released as vapor, thereby promoting Li depletion and surface oxidation of the LPSCl pellets [31, 43]. For this reason, 100°C was selected as the highest sintering temperature in this study.

Figure 2c,d presents the surface morphology, porosity, and pore size distribution of the SE pellets. Quantitative stereological analysis (Figure S6) shows that the surface porosity decreased from 12.9% in cold‐pressed LPSCl to 11.1%, 6.3%, and 3.2% for the 60°C, 80°C, and 100°C sintered pellets, respectively. Similarly, the average pore size was reduced from 0.34 µm to 0.31, 0.29, and 0.27 µm, with a corresponding decrease in standard deviation (from 0.19 to 0.14, 0.14, 0.12). Three images per SE pellet were analyzed for each sintered temperature to ensure reproducibility (Figure S7). Figure S8 summarized the effects of sintering temperature on the SE pellets surface porosity and bulk relative density. With increasing sintering temperature, surface porosity decreased, and the bulk relative density kept increasing from 84% in cold‐pressed LPSCl to 88.1% and 89.7% in 80°C and 100°C sintered pellets, respectively. Figure 2d provides the distribution of isolated pore areas on the pellet surface, categorized into four bins: 0.1‐0.2, 0.2‐0.4, 0.4‐0.6, and over 0.6 µm^2^. For cold‐pressed LPSCl, pores in the 0.1–0.2 µm^2^ range accounted for 44% of the total pore area, while pores over 0.6 µm^2^ comprised 19%. In the 80°C sintered LPSCl pellets, the percentage of pores of 0.1‐0.2 µm^2^ increased to 53%, and large pores over 0.6 µm^2^ decreased to 11%. For the 100°C sintered pellets, these values further shifted to 63% and 8%, respectively. This compaction of larger pores into smaller, more uniform pores upon sintering treatment is attributed to the lower hardness and creep behavior of LPSCl at elevated temperatures and pressure [31]. These results indicate that mild sintering under 50 MPa uniaxial press resulted in a more uniform surface with fewer voids and smaller pores compared to cold‐pressed LPSCl pellets, which is beneficial for mitigating lithium dendrite growth and penetration through SE [10, 23].

Additionally, Electrochemical Impedance Spectroscopy (EIS) measurements of SE pellets were conducted at 20 MPa. The recorded EIS spectra, and the corresponding Nyquist plots are presented in Figure 2e, with fitting using an equivalent circuit model shown in the inset. The high frequencies semi‐circle [∼0.5–7 MHz], assigned to the SE pellet resistance, was the largest for the cold‐pressed LPSCl. Increasing the sintering temperature progressively reduced the high frequency semi‐circle resistance, indicating improved SE ionic conductivity. As shown in Figure 2f, the SE resistance decreased from 450 Ω in cold‐pressed LPSCl to 400, 258, and 146 Ω in 60°C, 80°C, and 100°C sintered pellets, resulting in an increase of ionic conductivity of SE measured at 20 MPa from 0.58 mS cm^−1^ to 0.64, 0.96, 1.68 mS cm^−1^, respectively. This improvement is attributed to the denser bulk structure with fewer and smaller pores and uniform surface morphology [30].

However, the second semi‐circle at [∼ 10 kHz – 0.5 MHz], associated with the interfacial resistance between SE and stainless‐steel piston, exhibited the opposite trend. It increased with higher sintering temperatures, particularly for the 100°C, from 35 Ω (cold‐pressed) to 80, 135, 466 Ω in 60°C, 80°C, and 100°C sintered pellets, respectively. This sharp increase at 100°C is attributed to the surface chemical oxidation of the LPSCl pellets, as indicated by the XPS results, which suggests the formation of LiOH layer and a lithium depleted LPSCl surface with high resistance.

In summary, mild sintering at elevated temperature from 60°C to 100°C progressively improved the SE bulk density, reduced porosity, enhanced surface uniformity and increased ionic conductivity. However, excessive sintering in our particular set‐up, especially at 100°C, can induce SE surface oxidation that may impede Li‐ion transfer and affect lithium plating/ stripping behavior [44]. The impact of the SE mild sintering on lithium symmetric cell and full‐cell cycling performance will be discussed in the following sections.

### 2.2 LiF Surface Passivation of Lithium Metal

**FIGURE 3:**
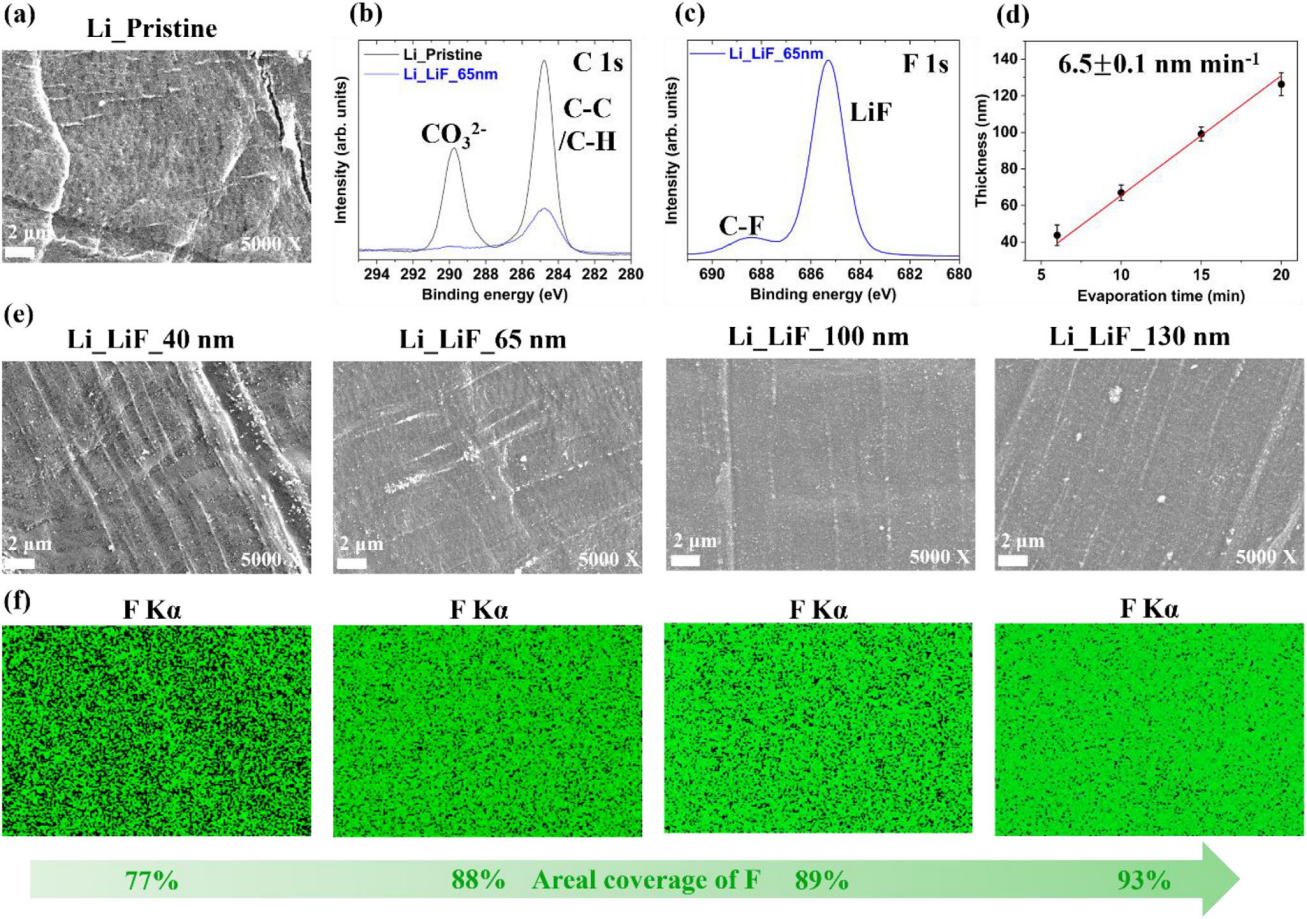
Characterization of lithium metal with and without LiF layer. (a) SEM plan‐view image of pristine Li surface morphology; (b) C 1s and (c) F 1s XPS core levels spectra acquired on pristine Li and Li surface coated with a 65 nm LiF layer; (d) relationship between the LiF layer thickness and evaporation time; (e) SEM plan‐view images comparing the surface morphology of Li coated with LiF layers of 40, 65, 100, 130 nm thickness; (f) EDX mapping of F Kα signal showing the areal coverage of F on Li surfaces with LiF layers of varying thickness.

In addition to the mild sintering treatment of the SE, the surface of the lithium metal foils was also modified by introducing a LiF passivation layer. As shown in Figure 3a, the scanning electron microscopy (SEM) image of pristine lithium metal exhibits noticeable rough surface with wrinkles, and hollows morphology, originating from the native passivation layer formed during manufacturing and calendaring in dry room. XPS analysis of the C 1s core level (Figure 3b) present peaks at 289.7 and 284.8 eV corresponding to lithium carbonate (Li_2_CO_3_) and C─H species, respectively, without excluding the presence of lithium hydroxide (LiOH). A series of LiF deposition layers with different thicknesses was prepared by varying the evaporation time. To estimate the LiF layer thickness, the lithium foil was bent to fracture the LiF coating and expose its cross‐section, which was then imaged by SEM. An example of 10 min LiF evaporation, corresponding to ∼65 nm thickness is shown in Figure S9. The presence of the LiF layer was further confirmed by Energy Dispersive X‐ray Spectroscopy (EDX) mapping of the F Kα. Three locations were examined using SEM cross‐sectional images for each LiF evaporation condition. The measured thicknesses, corresponding mean values, and standard deviations are summarized in Table S1. Evaporation times of 6 and 20 min exhibited larger standard deviations, whereas 10 and 15 min yielded improved uniformity. The correlation between the measured LiF thickness and evaporation time is shown in Figure 3d, with a fitted slope of 6.5 ± 0.1, which is determined as the LiF evaporation rate of 6.5 nm min^−1^. The in situ XPS of the F 1s core level (Figure 3c) shows a dominant peak at 685.3 eV, assigned to LiF, and a minor contribution at 688.4 eV attributed to C‐F species. The C 1s core level spectra (Figure 3b) exhibit strong attenuation of C─C/C─H and CO_3_^2−^ signals, confirming partial coverage on the native lithium surface by LiF. The SEM surface images (Figure 3e) indicate that a 40 nm LiF layer was insufficiently dense to fully cover the lithium surface. With increasing evaporation time, the LiF layer became denser and more uniform. The EDX mapping of F Kα (Figure 3f) demonstrated an increase in areal F coverage from 77% for the 40 nm layer to 88% for the 65 nm layer. Further increases in thickness did not significantly improve the fluorine surface coverage, indicating that evaporation beyond 15 min (corresponding to ∼100 nm thickness) mainly contributed to the LiF thickness growth rather than enhancing uniformity or density.

### 2.3 Electrochemical Measurements and Cycling Performance

#### 2.3.1 Galvanostatic Cycling of Li|LPSCl|Li Symmetric Cells

**FIGURE 4:**
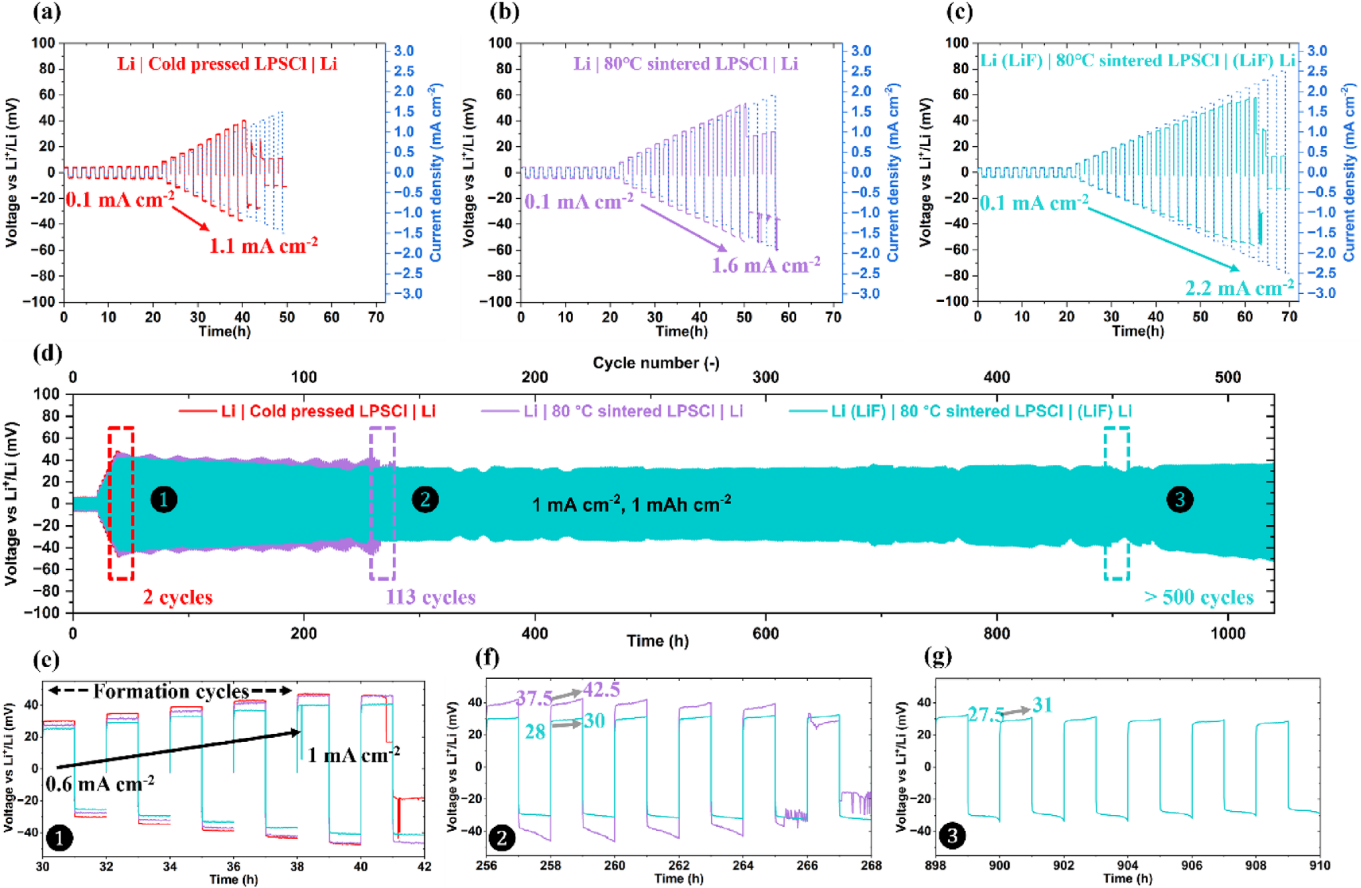
Cycling performance of lithium symmetric cells Li|LPSCl|Li. Critical current density of (a) pristine lithium with cold‐pressed LPSCl, (b) pristine lithium with 80°C‐sintered LPSCl, (c) lithium with LiF layer (65 nm) and 80°C sintered LPSCl, (d) Long‐term galvanostatic cycling stability at 1 mA cm^−2^ and 1 mAh cm^−2^, (e)(f)(g) voltage profiles corresponding to regions ❶❷❸ in (d).

To evaluate the impact of mild sintering of SE and the application of LiF passivation layer on lithium metal, galvanostatic cycling measurements were conducted on lithium symmetric cells. Each measurement started always with 10 formation cycles at 0.1 mA cm^−2^ and 0.1 mAh cm^−2^, following our standard protocol [42]. The critical current density (CCD) of cold‐pressed LPSCl with pristine Li (Figure 4a) was 1.1 mA cm^−2^, at which the cell short‐circuited. In contrast, after LPSCl sintering at 80°C for 6 h (Figure 4b), the CCD increased to 1.6 mA cm^−2^. As shown in Figure S10a,b, LPSCl sintering at 60°C and 100°C yielded CCDs of 1.3 and 1.4 mA cm^−2^, respectively. Although, both improved over the cold‐pressed LPSCl, the effect was less pronounced than the 80°C. Additionally, various sintering durations at 80°C were examined to assess the sensitivity of cycling performance to the sintering time. Compared with the cold‐pressed LPSCl, the CCD gradually increased to 1.2 mA cm^−2^ (Figure S10c), 1.4 mA cm^−2^ (Figure S10d), and 1.6 mA cm^−2^ (Figure 4b) after sintering for 1, 3, and 6 h, respectively. Extending the sintering time to 12 h did not further yield higher CCD value (Figure S10e). Therefore, a sintering time of 6 h was selected as the optimal condition in this study, ensuring sufficient densification of LPSCl pellets while avoiding unnecessary energy and time consumption.

These results indicate that the LPSCl sintering mitigates dendrite growth and delays short‐circuit by reducing the “percolation zones” and improving pellet density. Furthermore, the CCD performances of 80°C sintered LPSCl paired with 65 nm LiF‐coated lithium metal is depicted in Figure 4c, with comparisons for 40, 100, 130 nm LiF layers provided in Figure S11. Notably, only the 65 nm LiF layer provided a significant enhancement raising the CCD from 1.6 to 2.2 mA cm^−2^. In contrast, 40, 100, 130 nm LiF coatings resulted in CCD of 1.5, 1.3, 0.9 mA cm^−2^, respectively.

At a current density of 0.1 mA cm^−2^, the overpotential decreased from 5.0 mV for pristine lithium with cold‐pressed LPSCl to 4.4 mV for the 80 °C sintered pellet, reflecting improved ionic conductivity and higher pellet density. Introducing a 40 nm LiF coating on lithium metal maintained similar overpotential of 4.5 mV, while increasing the LiF thickness to 65 nm further reduced it to 3.7 mV, indicating enhanced interfacial chemical stability and improved contact between lithium and LPSCl pellet. However, 100 and 130 nm LiF coating resulted in higher overpotential of 5.6 and 6.6 mV, respectively, highlighting the detrimental effect of excessive LiF thickness. These findings confirm that 65 nm LiF coating provides optimal balance, ensuring sufficient coverage while avoiding excessive LiF thickness that would increase interface resistance due to its low ionic conductivity of 10^−11^ mS cm^−1^ [45]. Hereafter, Li (LiF) refers specifically to lithium metal with a 65 nm LiF coating.

The long‐term lithium plating and stripping behavior were also tested on Li symmetric cells Li|LPSCl|Li shown in Figure 4a–c: (Li|cold‐pressed LPSCl|Li), (Li|80°C sintered LPSCl|Li), and (Li (LiF)|80°C sintered LPSCl|(LiF) Li). At 0.5 mA cm^−2^ and 0.5 mAh cm^−2^, all three cells operated stably over 500 cycles under this mild condition (Figure S12). For comparison, the 100°C‐sintered LPSCl with pristine lithium metal (Figure S13a) exhibited voltage fluctuating and failed after 332 cycles. This aligns with the lower CCD observed (Figure S10b) and highlights the detrimental effect of interfacial instability caused by the higher sintering temperature.

The cycling stability of various LiF coating thicknesses combined with the 80°C sintered LPSCl was also assessed. The 100 nm LiF coating sustained over 500 cycles (Figure S13c), whereas the 40 and 130 nm LiF coatings (Figure S13b,d) failed after 467 and 18 cycles, respectively. Although the 100 nm LiF coating performed comparably to the 65 nm LiF in terms of cycle life, the latter consistently outperformed in CCD, overpotential, and cycling stability, confirming 65 nm as the optimal thickness.

Cycling at higher current density and areal capacity (1 mA cm^−2^, 1 mAh cm^−2^) was also evaluated (Figure 4d). The formation cycles were conducted, consisting of 10 cycles at a current density of 0.1 mA cm^−2^, followed by progressively increasing the current density from 0.1 to 1 mA cm^−2^ in steps of 0.1 mA cm^−2^. After these formation cycles, the cells were cycled at 1 mA cm^−2^ and 1 mAh cm^−2^ for long‐term stability testing. The cold‐pressed LPSCl with pristine lithium short‐circuited rapidly after only two cycles, whereas the 80°C sintered LPSCl extended cell lifetime to 113 cycles. A remarkable improvement was achieved by combining the 80°C sintered LPSCl with 65 nm LiF‐coated lithium, enabling stable cycling for over 500 cycles.

The zoomed‐in voltage profiles (Figure 4e–g) provide further insights. During the initial formation cycles “Region 1”, the potential evolution remained stable across the single plating and stripping process for all three cell configurations. Over time, however, the potential for the 80°C sintered LPSCl paired with pristine lithium gradually increased from 37.5 to 42.5 mV in “Region 2” (∼110th cycle), eventually leading to a short‐circuit failure. By contrast, the 65 nm LiF‐coating on lithium maintained a much more stable profile, with potentials from 28 to 30 mV in “Region 2” (∼110th cycles) and 27.5 to 31 mV in “Region 3” (∼430th cycles). This stability is attributed to more stable SEI between lithium and LPSCl pellets.

Taken together, both the CCD and long‐term cycling results confirm that the combination of 80°C sintered LPSCl with 65 nm LiF‐coated lithium significantly improves interfacial stability, enabling superior cycling performance and greatly extending cell lifetime.

#### 2.3.2 Impedance and Distribution of Relaxation Times Analysis of Li|LPSCl|Li Cells

To elucidate the mechanisms underlying the enhanced cycling performance, EIS spectra were compared and analyzed. The pre‐cycling impedance (Figure S14a), revealed clear differences among the various lithium symmetric cells configurations. Substituting cold‐pressed LPSCl with 80°C sintered LPSCl resulted in a pronounced leftward shift of the spectra to lower resistance, attributed to both the higher ionic conductivity of the sintered pellet (0.96 mS cm^−1^ vs. 0.58 mS cm^−1^ for cold‐pressed at 20 MPa) and improved interfacial contact between lithium and LPSCl. Furthermore, introducing a 65 nm LiF‐coated lithium further reduced the resistance, owing to the stable interface with the LPSCl and its uniform and dense LiF layer morphology. The influences of LiF coating thickness on impedance were also examined (Figure S14b), with all cells incorporating 80°C sintered LPSCl. A 45 nm LiF‐coated lithium increases the resistance, likely due to the non‐uniform coverage of LiF. Increasing the LiF thickness to 65 nm significantly lowered the resistance, indicating improved interfacial contact and chemical stability. However, further increases to 100 and 130 nm did not yield additional improvements, instead, the resistance surpassed that of pristine lithium. These observations align with the cycling performance presented in Figures S11–S13, underscoring the critical role of LiF layer thickness and uniformity, confirming 65 nm LiF as the optimal thickness.

The EIS evolution during cycling were conducted every 50 cycles during lithium plating and stripping at 0.5 mA cm^−2^ and 0.5 mAh cm^−2^ (cycling shown in Figure S12). To visualize the underlying kinetic processes and enable quantitative comparisons, Distribution of Relaxation Times (DRT) analysis was employed [46, 47]. The data processing procedure is illustrated in Figure S15 and described in detail in the subsequent **Note** in the Supporting Information. As shown in Figure S16, the impedance spectra reproduced from the computed DRT patterns aligned closely with the experimental data for all three cells, validating the accuracy and reliability of the DRT analysis for further qualitative and quantitative assessments.

**FIGURE 5:**
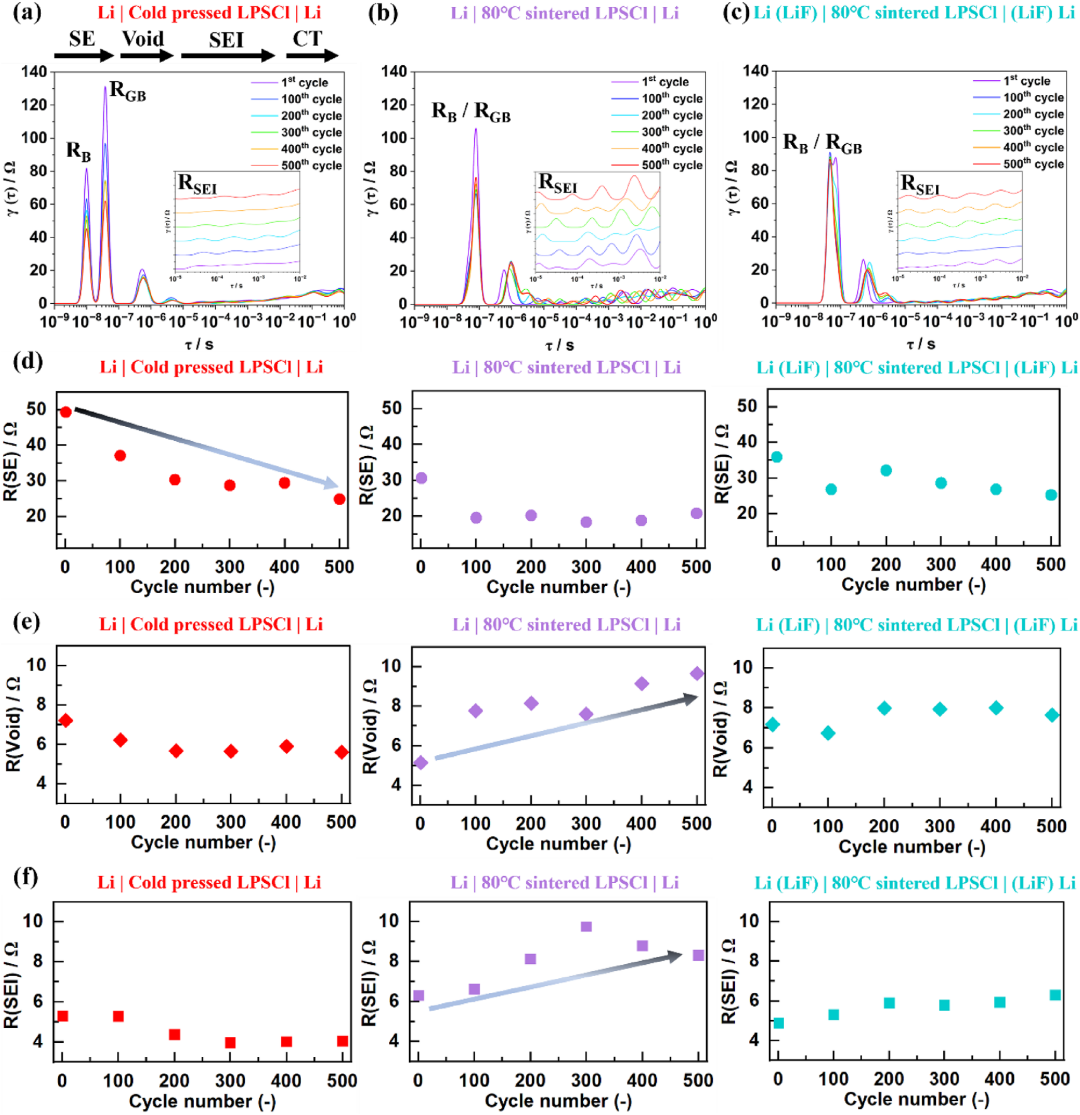
The Distribution of Relaxation Times (DRT) analysis of Li|LPSCl|Li symmetric cells during cycling at 0.5 mA cm^−2^ and 0.5 mAh cm^−2^. Evolution of DRT patterns depicted in line shape, (a) pristine lithium with cold‐pressed LPSCl, (b) pristine lithium with 80°C sintered LPSCl, (c) 65 nm LiF‐coated lithium with 80°C sintered LPSCl. Evolution of different resistances with cycle number, (d) resistance of solid electrolyte (SE), (e) resistance of void, (f) resistance of SEI.

The evolution of DRT patterns from first to 500^th^ cycle is summarized in Figure 5a–c. Based on the distinct time constants of peaks, four regimes were categorized, corresponding to typical kinetic processes in lithium metal ASSBs [46]. The peaks with time constants smaller than ∼10^−7^ s are attributed to the response of the LPSCl separator (R_SE_), including contributions from bulk (R_B_) and grain boundary (R_GB_) resistances, consistent with literature report [48]. The time constants between 10^−5^ and 10^−2^ s represent the SEI with lithium metal (R_SEI_) [49]. The intermediate region between R_SE_ and R_SEI_ is attributed to void‐related impedance (R_Void_) [50]. The 10^−2^ to 10^0^ s range reflects charge transfer resistance associated with lithium plating and stripping reactions. The contour maps of DRT patterns are provided in Figure S17. For the cold pressed LPSCl, two distinct peaks associated with R_B_ and R_GB_ appeared in the R_SE_ region, initially at around 80 and 130 Ω, respectively. In contrast, for the 80°C sintered LPSCl, the R_B_ and R_GB_ peaks were merged into a broad peak. This was attributed to the denser and more uniform structure of the sintered LPSCl pellet, which enhanced the transport behavior of Li ions across SE and thereby reduced the R_GB_ and R_B_, as corroborated by the left‐down shift of impedance to lower resistance at 7 MHz in Figure S16 and the higher ionic conductivity [48]. In the SEI‐related time domain, very minor changes were observed for the cold‐pressed LPSCl. However, for the 80°C sintered LPSCl paired with pristine lithium, pronounced fluctuations and the emergence of new peaks were evident in the DRT patterns. A closer inspection of the zoomed‐in inset in Figure 5b and the highlighted white rectangle in Figure S17b reveals these features, suggesting the presence of dynamic interfacial processes induced by the better interface contact between the lithium and LPSCl which enhance the SEI signal. Upon introducing a 65 nm LiF‐coated lithium, the evolution of the R_SEI_ became significantly more moderate indicating partial mitigation of the SEI formation.

To quantitatively analyze the evolution of individual electrochemical processes, the integrated peak areas within the pre‐defined regimes, corresponding to the respective resistances, were calculated and compared [47]. As shown in Figure 5d, the initial R_SE_ of the cold pressed LPSCl paired with pristine lithium was 49.3 Ω, which continuously decreased by 49.7% to 24.8 Ω after 500 cycles. This reduction in R_SE_ was attributed to the growth and penetration of lithium dendrite through SE, which served as the preferential plating and stripping sites and shortened the ionic transport distance [51]. In contrast, the 80°C sintered LPSCl exhibited a significantly lower initial R_SE_ of 30.6 Ω due to its denser microstructure. Although it slightly decreased after 100 cycles, it then stabilized, reaching a final value of 20.7 Ω, corresponding to a 32.3% reduction. With the introduction of a LiF interfacial layer, the reduction in R_SE_ after 500 cycles was further moderated to 29.5% (from 35.9 to 25.3 Ω). This relatively smaller decline in R_SE_ highlights the effectiveness of both sintering treatment and LiF protection in suppressing lithium dendrite formation and penetration.

For the SEI resistance (Figure 5f), a notable increase from 6.3 Ω to a peak value of 9.8 Ω was observed for the 80°C sintered LPSCl paired with pristine lithium. This increase was attributed to the enlarged contact area between lithium and LPSCl, as well as the elevated interfacial resistance introduced by the sintering treatment (as discussed in Figure 2). In parallel, continuous SEI growth during cycling led to unstable contact at the Li/SE interface, resulting in an increase of R_Void_ from 5.1 to 9.6 Ω (Figure 5e). However, when the 80°C sintered LPSCl was paired with 65 nm LiF‐coated lithium, improved interfacial stability was achieved. The evolution of both R_SEI_ (from 4.9 to 6.3 Ω) and R_Void_ (from 7.2 to 7.6 Ω) became more stable, demonstrating that LiF effectively mitigated interfacial reactions between lithium and LPSCl, while enhancing and stabilizing interfacial contact.

Furthermore, the stability comparison between 80°C and 100°C sintered LPSCl was investigated. The DRT analysis (Figure S18) was conducted for Li|LPSCl|Li symmetric cell employing 100°C‐sintered LPSCl during cycling, corresponding to the cycling performance shown in Figure S13a. From the DRT patterns evolution in Figure S18a, the peaks associated with R_SE_ continuously decreased, similar to the behavior observed for cold‐pressed LPSCl (Figure 5a). The peaks corresponding to R_Void_ exhibited substantial signals compared with those of 80°C sintered LPSCl (Figure 5b). Quantitatively, as presented in Figure S18b, the R_SE_ of 100°C‐sintered LPSCl decreased from 28.9 to 8.8 Ω after 300 cycles, representing a 69.5% reduction, which was significantly larger than that of the 80°C sintered LPSCl (32.3% reduction after 500 cycles). From Figure S18c, the R_Void_ of 100°C‐sintered LPSCl increased from 12.5 to 25.2 Ω after 200 cycles, whereas that of the 80°C sintered LPSCl increased from 5.1 to 8.1 Ω. The R_SEI_ (Figure S18d) for 100°C‐sintered LPSCl increased from 7.4 to 8.9 Ω after 100 cycles, higher than the increase from 6.3 to 6.6 Ω observed for the 80°C sintered LPSCl. To summarize, the pronounced decline in R_SE_ indicates more severe lithium dendrite growth with 100°C‐sintered LPSCl. Meanwhile, the higher initial values and stronger increasing trends in R_Void_ and R_SEI_ demonstrate significant void formation and inferior interfacial stability, ultimately leading to short circuit after 332 cycles. These DRT results are consistent with the surface oxidation of LPSCl observed after sintering at 100°C, and with its poorer cycling performance. Therefore, 80°C was confirmed as the optimal sintering temperature in this study.

#### 2.3.3 Coulometric Titration Time Analysis of Li|LPSCl|SS Asymmetric Cells

**FIGURE 6:**
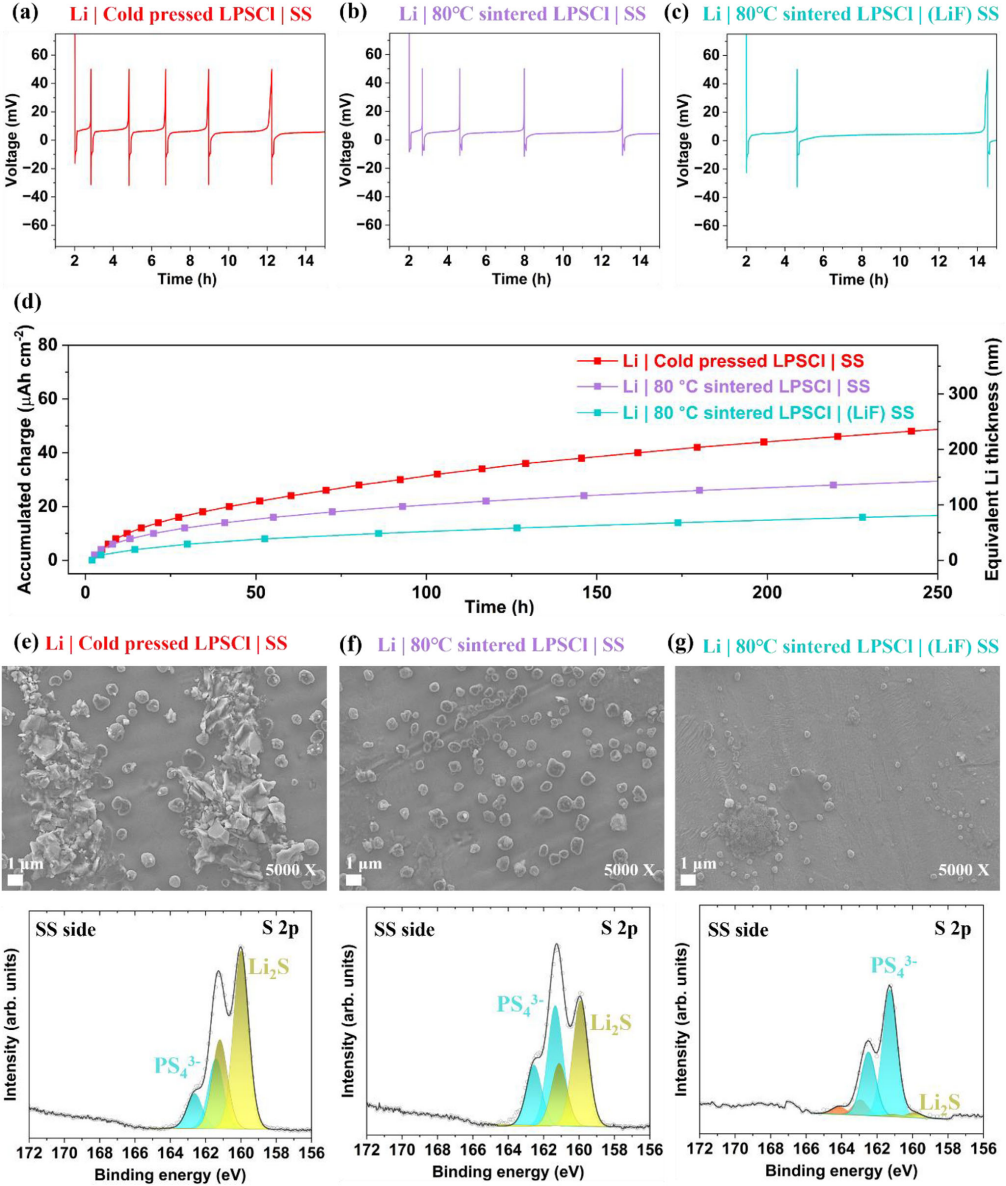
Solid electrolyte interface (SEI) characterization via CTTA. Voltage profiles within 15 h of (a) pristine lithium with cold‐pressed LPSCl and SS, (b) pristine lithium with 80°C sintered LPSCl and SS, (c) pristine lithium with 80°C sintered LPSCl and 65 nm LiF‐coated SS. (d) sum of accumulated charge over time. (e)(f)(g) SEM images and S 2p XPS spectra acquired on SS sides after CTTA experiments, corresponding to configurations of (a)(b)(c).

The coulometric titration time analysis (CTTA) was further conducted to investigate SEI formation in asymmetric Li|LPSCl|stainless steel (SS) cells [22, 52]. The cycling protocol involved continuously plating 2 µAh cm^−2^ of lithium on SS and resting at OCV status until complete lithium consumption into the SEI, defined by a cut‐off voltage of 50 mV. The corresponding voltage profiles within 250 h are shown in Figure S19. From the zoomed‐in voltage profiles of the initial stages within 15 h (Figure 6a–c), the cold‐pressed LPSCl required 0.75 h to consume all the plated lithium in SEI. In comparison, the 80°C sintered LPSCl required a shorter time of 0.6 h, due to its more uniform surface morphology. Notably, the presence of a 65 nm thick LiF layer, serving as barrier layer, significantly delayed the plated lithium conversion into SEI, extending the process to 2.5 h. As the cycling progressed, the accumulated charge consumption over time was calculated and reported in Figure 6d. The fastest lithium consumption and SEI formation occurred in the configuration of cold‐pressed LPSCl with SS, likely due to more hotspots for lithium to deposit and lithium dendrite growth, which increased the local surface area of lithium available for reaction with LPSCl [22]. By contrast, the 80°C sintered LPSCl, with smaller and fewer voids on the surface, mitigated the dendrite formation and penetration, therefore slowed down the consumption rate. The most stable interface was observed for the system combining 80°C sintered LPSCl with a 65 nm LiF‐coated lithium, where LiF acted as a barrier layer, effectively suppressing the lithium consumption and undesired side reactions with LPSCl.

Following 250 h of cycling, the cells were disassembled for post‐mortem analysis. The SS was separated from the SE pellet, and both sides were investigated via SEM and XPS. The SS side was used to assess the morphology of the SEI. On the LPSCl pellet sides (Figure S20), the surface morphology confirms the improved surface uniformity and increased density of the LPSCl pellets following mild sintering treatment. On the SS sides, as shown in Figure 6e–g and Figure S21, numerous spherically shaped particles were observed on the surface of SS in the cold‐pressed LPSCl sample, consistent with the findings reported by Janek and co‐workers [52]. These particles are identified as SEI components formed during CTTA. The larger, irregularly shaped particles are fractured LPSCl fragments remaining on the SS after the separation. In some regions, the whisker‐shaped clusters were observed, indicative of localized lithium deposition and dendrite growth. For the 80°C sintered LPSCl sample, the number of spherical particles was decreased, and lithium whiskers were no longer evident, suggesting reduced cumulative charge consumption and effective prohibition of lithium dendrite growth. When 80°C sintered LPSCl was paired with 65 nm LiF‐coated SS, the number and size of particles further decreased, consistent with the slowest SEI formation rate and the most stable interface.

The S 2p XPS spectra on SS sides (Figure 6e–g) of cold pressed LPSCl sample revealed substantial Li_2_S formation confirmed by a strong peak located at 160 eV, which is a degradation reduction byproduct of LPSCl and deemed a non‐beneficial SEI component due to its non‐conductive properties [16]. With 80°C sintered LPSCl, the intensity of the Li_2_S peak was reduced but still evident. In contrast, the S 2p spectra of the LiF coated SS was dominated by PS_4_^3−^ units in LPSCl with only minor Li_2_S presence, indicating significant alleviation of (electro‐)chemical reactions between the plated lithium and LPSCl. On the LPSCl pellet sides (Figure S20), the Li_2_S content trend was consistent with that observed on SS sides. Furthermore, the high interfacial energy of LiF with lithium (73.28 meV Å^−2^) as reported by Wang et al. [53], compared to normal SEI components such as Li_2_S (19.01 meV Å^−2^) and LiCl (37.55 meV Å^−2^), contributes to its superior dendrite‐suppressing ability. The differences in SEI formation behavior among the cells, as revealed by CTTA analysis, correlate well with the evolution of R_SE_, R_Void_, and R_SEI_ during cycling, as determined from DRT analysis.

To summarize, owing to the electron‐blocking effect of LiF with its low electronic conductivity (10^−10^ S cm^−1^) [54, 55], the decomposition of LPSCl and SEI formation was effectively mitigated. Together with the effect of high interfacial energy with lithium, incorporating a uniform LiF passivation layer significantly enhances interfacial stability and suppresses the lithium dendrite formation.

#### 2.3.4 Galvanostatic Cycling of NCM811|LPSCl|Li Full Cells

**FIGURE 7:**
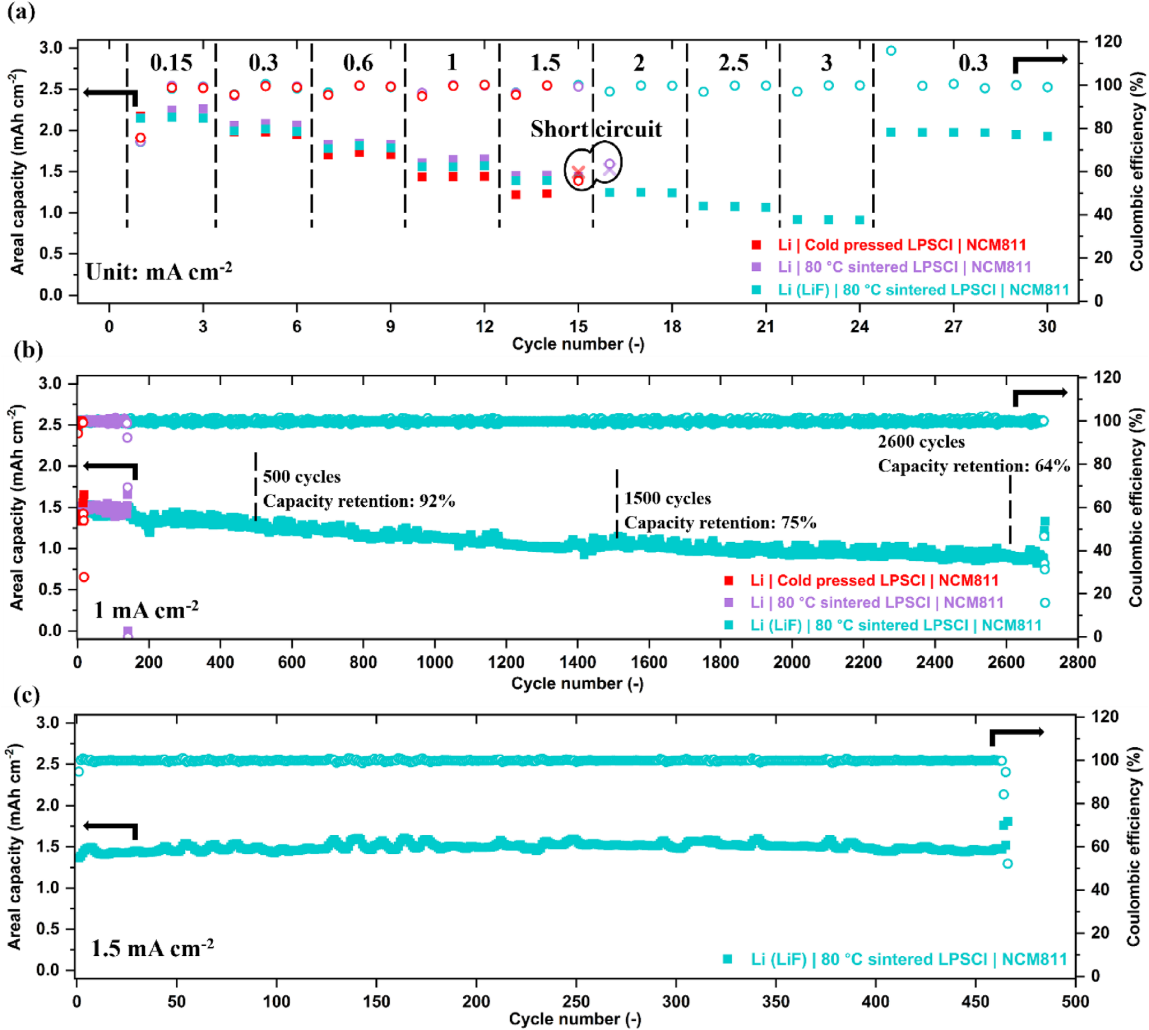
Cycling performance of NCM811|LPSCl|Li full cells corresponding to pristine lithium with cold‐pressed LPSCl, pristine lithium with 80°C‐sintered LPSCl, and 65 nm LiF‐coated lithium with 80°C‐sintered LPSCl. (a) rate capability test, (b) long‐term cycling at 1 mA cm^−2^, (c) long‐term cycling at 1.5 mA cm^−2^.

The cycling performance of full cells employing an NCM811 positive electrode was evaluated and presented in Figure 7a. The rate capability tests conducted by applying 3 cycles at different charge and discharge rates, ranging from C/20 to 1C, equivalent to current densities from 0.15 to 3 mA cm^−2^. The associated voltage profiles are depicted in Figure S22 for the three cell configurations, pristine lithium with cold‐pressed LPSCl, pristine lithium with 80°C sintered LPSCl, and 65 nm LiF‐coated lithium with 80°C sintered LPSCl. When the cycling rate was increased to C/2 (1.5 mA cm^−2^), the cell with pristine lithium and cold‐pressed LPSCl was shortened, as evidenced by a significant drop of coulombic efficiency (CE) and abnormal voltage fluctuating (Figure S22a). The cell with 80°C sintered LPSCl with pristine lithium sustained at 1.5 mA cm^−2^ but was shorted quickly during the first cycle at 2 mA cm^−2^ (Figure S22b). In contrast, the excellent full cell rate capability was achieved when 80°C treated LPSCl paired with 65 nm LiF‐coated lithium, consistent with the improvements observed in lithium symmetric cells. This configuration maintained stable charge and discharge voltage profile even at 1C (3 mA cm^−2^), delivering an areal capacity of 0.9 mAh cm^−2^ and a CE of 97%. Upon reducing the cycling rate back to C/10 (0.3 mA cm^−2^), the areal capacity recovered to 1.98 mAh cm^−2^, comparable to the initial value of 1.99 mAh cm^−2^ at C/10.

The long‐term cycling measurements further demonstrates the enhancement in cycling stability. Figure 7b compares the performance of three different cell configurations cycled at C/3, equivalent to a current density of 1 mA cm^−2^. The initial areal capacity for pristine lithium with cold‐pressed LPSCl was 1.36 mAh cm^−2^, and the short‐circuit occurred after 16 cycles. Owing to the improved surface uniformity and density of 80°C sintered LPSCl pellet, the lifetime was extended to 139 cycles with a higher initial areal capacity of 1.49 mAh cm^−2^. By coupling 80°C sintered LPSCl with a 65 nm LiF‐coated lithium metal, the full cell exhibited an exceptionally long lifetime exceeding 2700 cycles, with an initial areal capacity of 1.46 mAh cm^−2^. Notably, the capacity retention was 92% after 500 cycles and 75% after 1500 cycles. From Figure 6d, the accumulated charge of CTTA associated with SEI formation within 250 h was 16 µAh cm^−2^, corresponding to a consumed lithium equivalent thickness of 77.6 nm. Extrapolating this formation rate to 1500 cycles at 1 mA cm^−2^ yields an estimated active lithium loss of approximately 1.4 µm, indicating that only 2.8% of the 50 µm lithium metal anode becomes electrochemically inactive. Therefore, given the stable interface between LiF‐coated lithium anode and the 80°C sintered solid electrolyte, together with the ample lithium reservoir (48.6 µm) confirmed by both DRT and CTTA analysis, the progressive slow fading of the full cell is primarily attributed to degradation on the cathode side. For uncoated NCM811, the (electro‐)chemical instability at the cathode/SE interface leads to the formation of inactive and resistive interphases, as well as potential physical disconnection among particles in the composite cathode, thereby increasing resistance and accelerating capacity decay after long‐term cycling [56].

To evaluate the cell ability to sustain against harsh cycling condition at high current density and to recover the capacity after long‐term, we performed a C‐rate test after 500 cycles at C/3 (Figure S23) by increasing gradually the cycling rate from C/3 to 1C (3 mA cm^−2^), then decreased to C/10 and C/5, and finally held at C/3 for an additional 500 cycles. From Figure S23, the areal capacity declined from 1.43 to 1.29 mAh cm^−2^ after 500 cycles, corresponding to capacity retention of 90%, demonstrating excellent reproducibility in comparison with the results shown in Figure 7b. After the high C‐rate cycling phase up to 3 mA cm^−2^ without short‐circuit and subsequent return to C/3, the areal capacity initially dropped to 1.17 mAh cm^−2^, but gradually recovered to 1.29 after 62 cycles. Following further 500 cycles at 1 mA cm^−2^, the areal capacity remained at 1.19 mAh cm^−2^, indicating outstanding cycling stability and capacity recovery. Additionally, at a higher current density of 1.5 mA cm^−2^, as shown in Figure 7c, the full cell with 80°C sintered LPSCl and 65 nm LiF‐coated lithium metal maintained stable cycling performance over 450 cycles, with an initial areal capacity of 1.36 mAh cm^−2^ and an average CE of 99.8%.

**FIGURE 8:**
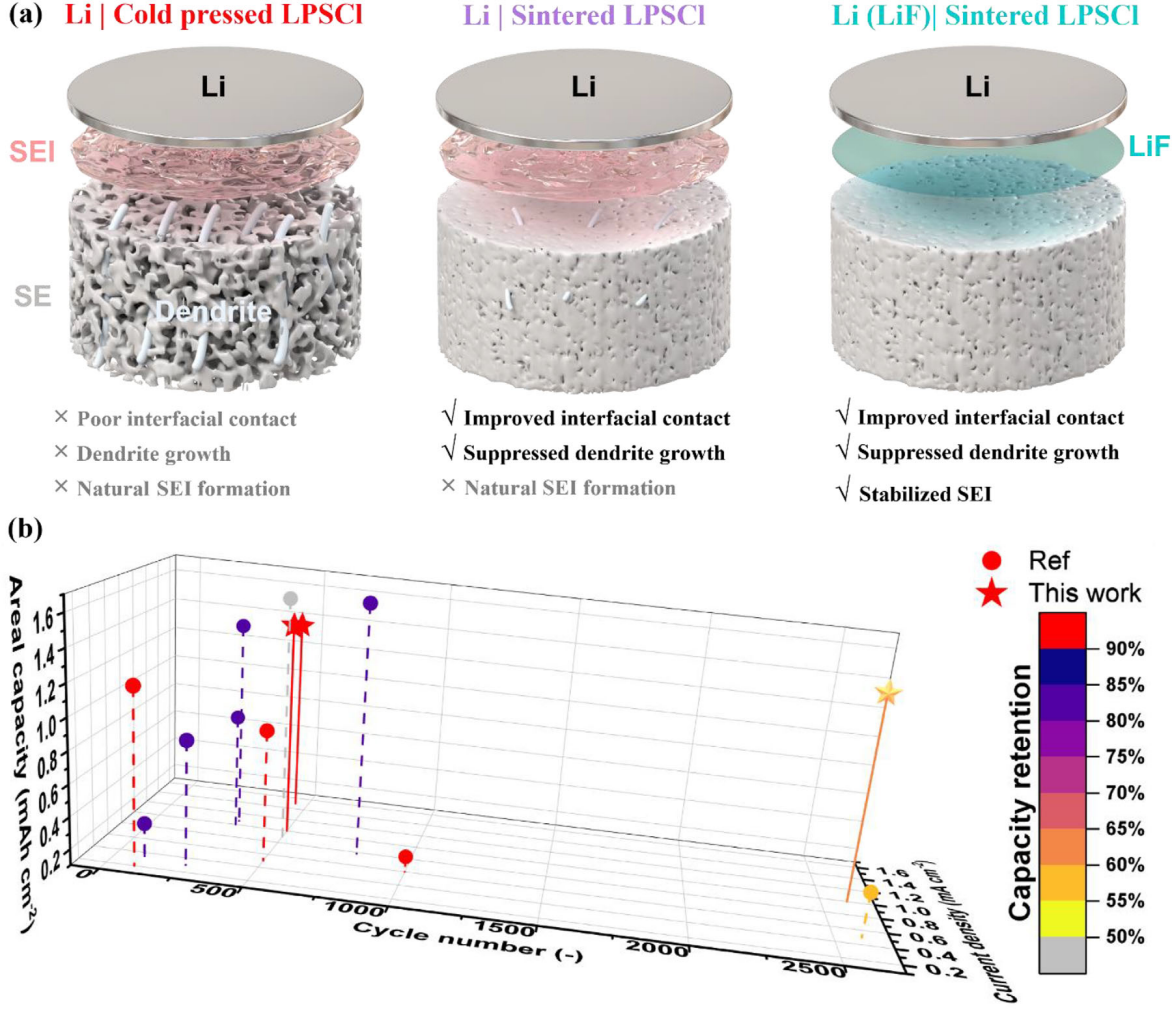
(a) Schematic illustrations of the improvements achieved by mild sintering of SE pellets and LiF passivation of lithium metal. (b) Comparison of full cell cycling performance with reported results in reference, considering current density, areal capacity, cycle number, and capacity retention.

Based on the above discussion, the underlying mechanisms for cycling performance improvement are illustrated (Figure 8a). In cold‐pressed SE, the abundance of irregular pores leads to poor interfacial contact with Li metal, which promotes dendrite nucleation and facilitates their rapid propagation through the low‐density and highly porous SE pellet, ultimately resulting in short circuit. The sintering treatment densifies the SE surface and enhances bulk density, thereby improving Li/SE contact and suppressing dendrite penetration. However, this process also enlarges the interfacial area, accelerating SEI formation and gradually compromising interfacial stability. The further introduction of a uniform LiF passivation layer on Li metal effectively prevents interfacial side reactions, mitigates uncontrolled SEI growth, and thus provides a synergistic pathway to stabilize the Li/SE interface and inhibit dendrite growth. Consequently, superior cycling performance was demonstrated for lithium metal full cells compared to literature reports (Figure 8b). The synergistic strategy supports stable long‐term cycling with sustained capacity retention, even at high current density and areal capacity. The references selected for comparison were restricted to studies employing sulfide‐based solid electrolytes and lithium metal anodes (details in Table S2).

## 3 Conclusion

In summary, we have introduced a synergistic strategy combining mild sintering of the LPSCl SE and 65 nm LiF‐coated lithium metal to suppress lithium dendrite growth and stabilize the interface between lithium and LPSCl in ASSBs. Sintering at an optimized temperature of 80°C with a pressure of 50 MPa enhances the surface uniformity of LPSCl pellets by reducing porosity, thereby improving ionic conductivity, and preventing lithium dendrite penetration. The 65 nm LiF passivation layer with uniform coverage deposited on lithium metal improves interfacial contact and stabilize SEI formation. Remarkably, the combined approach delivers substantially superior performance in both lithium symmetric cells (critical current density of 2.2 mA cm^−2^) and lithium full cells employing a LiNi_0.8_Co_0.1_Mn_0.1_O_2_ cathode (over 2700 cycles at C/3, 1 mA cm^−2^) at room temperature. Supported by comprehensive analyses, these findings underscore the pivotal role of solid electrolyte separator quality and interfacial stability in improving the cycling performance of lithium metal in ASSBs.

## 4 Experimental Section

### 4.1 Materials

The solid electrolyte (SE) Li_6_PS_5_Cl (LPSCl) (3 µm particle size) was purchased from INCHEMS Co., Ltd. The 50 µm thick lithium metal coated on copper foil (13 µm thick) was obtained from China Energy Lithium Co., Ltd. The LiNi_0.8_Co_0.1_Mn_0.1_O_2_ (NCM811) cathode material was sourced from MSE, and the conductive carbon additive C65 was from Imerys. The LPSCl powder was ball milled before pressing into pellets. The following condition was applied: 5 ZrO_2_ balls (10 mm in diameter) with 500 mg LPSCl powder in a FRITSCH Planetary Micro Mill at 140 rpm for 1 h. The cathode composite, consisting of NCM811 used as received without any surface coating, LPSCl, and C65 in a mass ratio of 70:27:3, was prepared by hand‐mixing in a mortar. The lithium Fluoride (LiF) powder was purchased from Sigma‐Aldrich and filled into the crucible made of graphite for electron beam evaporation. The electron beam source operated at an acceleration voltage of 3 kV and a current of 35 mA, within a vacuum chamber maintained at 3.5 × 10^−6^ mbar. The lithium foils are transferred between the glovebox and the ultra‐high vacuum system for surface passivation and XPS measurements under a controlled atmosphere using specific transfer chamber, preventing any surface modification from exposure to air or moisture. All materials synthesis, electrode preparation, cell assembly, and electrochemical cycling were conducted in an Argon‐filled glovebox at room temperature.

### 4.2 Cell Assembly and Electrochemical Measurements

Bulk‐type ASSBs were assembled into custom‐made cell following the cell assembly details described in our previous work [42]. For the lithium symmetric cells, 60 mg LPSCl was loaded into a 7 mm diameter die and compacted using a hydraulic press at 380 MPa for 1 min. When the sintering treatment applied, the SE pellets were transferred within the die under 50 MPa into the vacuum chamber (∼5.10^−1^ mbar) connected to the glovebox for sintering at various temperature (60°C, 80°C, and 100°C) for 6 h. A lithium edge protection was used to achieve reliable cycling following the recommendation reported in our previous work [42]. The edge is made of an insulating polymer ring (high‐density polyethylene: HDPE) with an outer diameter of 7 mm and an inner diameter of 5 mm pressed on both sides of SE pellets at 25 MPa. The 50 µm lithium metal was punched into 5 mm discs and pressed onto SE pellets at 25 MPa for 1 min. For the NCM811| LPSCl | Li full cells, 4.2 mg cathode composite (7.6 mg (NCM811) cm^−2^) was loaded and pressed onto SE pellets at 510 MPa for 1 min. The anode side was assembled following the same procedure as the symmetric cells. Subsequently, the assembled cells were closed at ∼20 MPa (calibrated using an integrated pressure sensor) [42], rested at open circuit voltage (OCV) for 2 h, and cycled inside the glovebox.

Galvanostatic cycling measurements were conducted on lithium symmetric cells at different current densities and areal capacities. Full cells were cycled at various C‐rates between 2.7 and 4.3 V vs. Li^+^/Li. A theoretical capacity of 200 mAh g^−1^ was used to calculate the applied current density, resulting in 1 C equivalent to 3 mA cm^−2^ on the lithium metal anode. Electrochemical impedance spectroscopy (EIS) was performed using a Biologic VSP‐300 potentiostat in the frequency range from 10 mHz to 7 MHz with 10 mV amplitude. The Distribution of Relaxation Times (DRT) analysis was performed using RelaxIS software (rhd instruments) to transform the EIS data from the frequency domain into the time domain. The detailed procedure of the DRT processing is provided in the Note section accompanying Figure S15 in the supplementary information.

The ionic conductivity was calculated with the pellet thickness t (cm), resistance R (Ω), and area A (cm^−2^): $\sigma =\frac{t}{R\times A}$.

Coulometric Titration Time Analysis (CTTA) was conducted using a stainless steel (SS) disk as working electrode and lithium metal as counter electrode. The SS disk (7 mm in diameter) was pressed on the SE pellets at 125 MPa to ensure appropriate contact. Lithium metal foil (5 mm in diameter) was placed in the center of pellets and pressed at 25 MPa. The cell was closed at 20 MPa and rested for 2 h. In each titration step, 2 µAh cm^−2^ lithium was plated onto the SS electrode, followed by an OCV hold until the voltage exceeded 50 mV, indicating complete lithium consumption. Upon reaching this threshold, a new titration step commenced. This cycle was repeated continuously for a total duration of 250 h. Finally, the cells were disassembled, and the SS electrode was easily separated from the SE pellets for further characterization.

To prevent air exposure and surface chemistry modifications, all transfers from the glovebox to the different ultra‐high‐vacuum (UHV) analytical methods described below were conducted using a dedicated air‐tight transfer chamber.

### 4.3 Characterization Methods

1. X‐ray photoelectron spectroscopy (XPS) measurements were conducted on a VG ESCALAB 220iXL spectrometer (Thermo Fisher Scientific) using focused monochromatized Al Kα radiation (1486.6 eV) with a beam size of ∼500 µm^2^ (power of 150 W). The spectrometer was pre‐calibrated by performing a measurement on a clean silver surface, whereby the Ag 3d_5/2_ peak was aligned to a binding energy of 368.25 eV with a full width at half‐maximum of 0.78 eV at a pass energy of 20 eV. Survey spectra were recorded with a dwell time of 50 ms, using a pass energy of 50 eV in steps of 0.5 eV. For high‐resolution spectra, these parameters were adjusted to 20 and 0.05 eV, respectively. Peak deconvolution was performed using CasaXPS software, applying the sum of Gaussian (70%) and Lorentzian (30%) line shapes after a Shirley‐type background subtraction. The spin‐orbit splitting energy (ΔE) and branching ratio (β) applied for the deconvolution of the S 2p_3/2+1/2_ and P 2p_3/2+1/2_ were fixed to ΔE_S2p_ = 1.2 eV, β_S2p_ = 0.5, ΔE_P2p_ = 0.87 eV, and β_P2p_ = 0.5. No charge compensation was applied, and the binding energy calibration was referenced to the C 1s core level located at 284.8 eV.
2. Scanning electron microscopy (SEM) measurements were conducted in a field emission gun equipped SEM Zeiss ULTRA 55 using the Secondary Electron Emission Detector of 5 kV accelerating voltages and ∼5 mm working distance. Energy‐dispersive X‐ray spectroscopy (EDX) mapping was conducted at 5 kV using an Oxford Ultim Extreme detector, with data processing performed in AZtec software. The EDX maps were recorded at a resolution of 1024 × 768 pixels. To estimate the LiF layer thickness, the lithium foil was bent to induce cracks in the LiF layer, allowing it to be separated from the metallic lithium and its cross‐section observed in SEM images.
3. The powder X‐ray diffraction (PXRD) samples were flame‐sealed under Ar atmosphere in a borosilicate capillary with a diameter of 0.5 mm. The PXRD patterns were collected between a 2Θ angle of 10° and 90° with a step size of 0.0167° using a PANalytical Empyrean Series 2 diffractometer equipped with a Cu Kα source (λ = 1.541 Å) and with a linear X'Celerator detector.

## Funding

This work was supported by Swiss National Science Foundation (SNSF) (Grant No. Sinergia CRSII5_202296), “LiBaCoat” project supported by Hightech Zentrum Aargau AG and Forschungsfonds Aargau, and Nano‐Argovia “BatCoat” project from Swiss Nanoscience Institute (SNI).

## Conflicts of Interest

The authors declare no conflicts of interest.

## Supporting information




**Supporting File**: advs73495‐sup‐0001‐SuppMat.docx.

## Data Availability

The data that support the findings of this study are openly available in Zenodo at https://doi.org/10.5281/zenodo.17094734, reference number 17094734.
