# Supplementary material for: Synergistic Effects of Solid Electrolyte Mild Sintering and Lithium Surface Passivation for Enhanced Lithium Metal Cycling in All‐Solid‐State Batteries
Source: Adv Sci (Weinh). 2026 Jan 8;13(13):e21791. doi: 10.1002/advs.202521791 (PMC12955938; doi:10.1002/advs.202521791)
Supplement: Supplementary file 1 — Supporting File: advs73495‐sup‐0001‐SuppMat.docx. [file ADVS-13-e21791-s001.docx]

Supporting Information

**Synergistic Effects of Solid Electrolyte Mild Sintering and Lithium Surface Passivation for Enhanced Lithium Metal Cycling in All-Solid-State Batteries**

Jinsong Zhang, Robin N. Wullich, Thomas J. Schmidt, Mario El Kazzi*

J. Zhang, R. N. Wullich, T. J. Schmidt, M. El Kazzi

PSI Center for Energy and Environmental Sciences, CH-5232 Villigen PSI, Switzerland

E-mail: [mario.el-kazzi@psi.ch](mailto:mario.el-kazzi@psi.ch)

T. J. Schmidt
Institute for Molecular Physical Science, ETH Zurich, CH-8093 Zurich, Switzerland


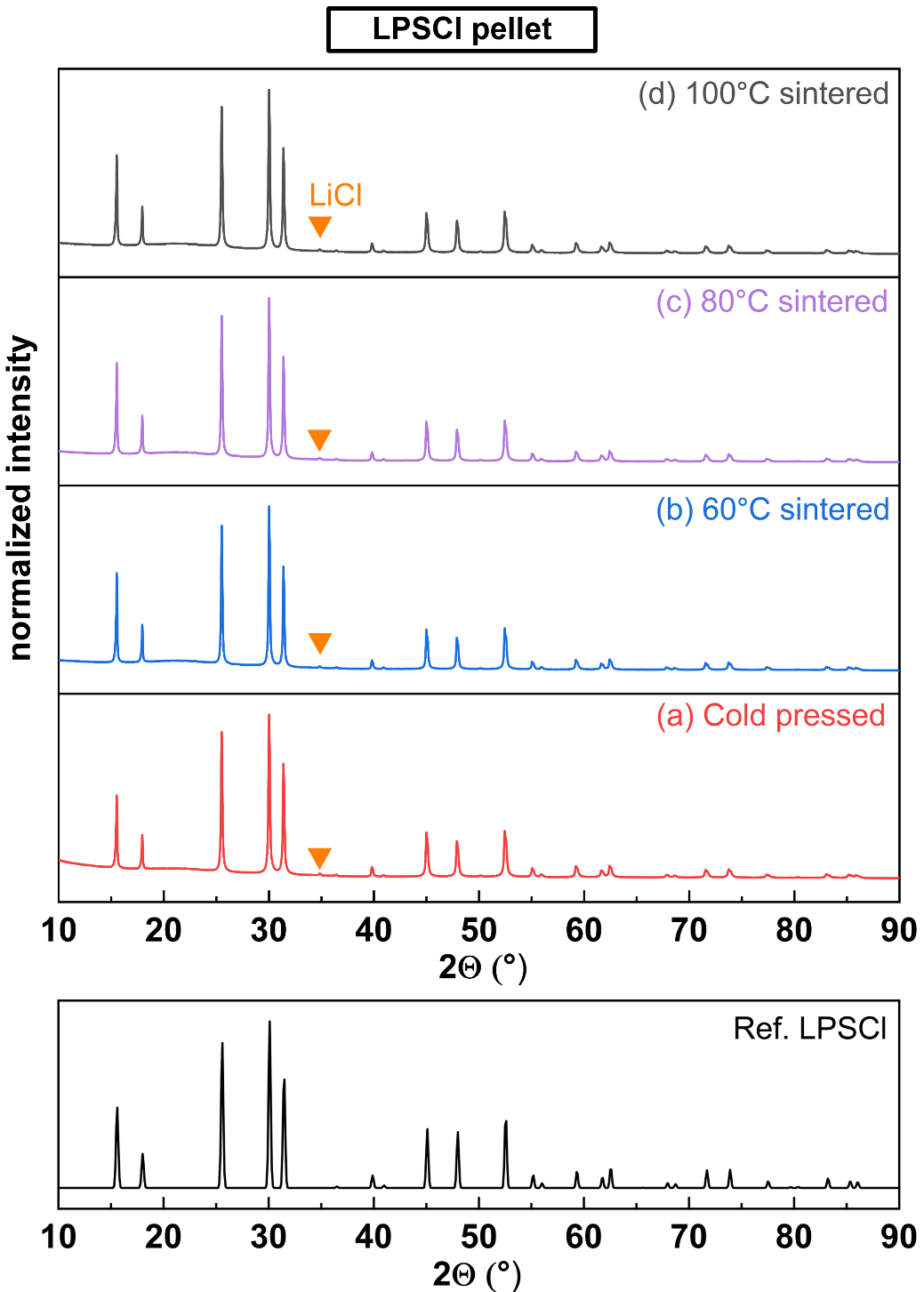


Figure_S 1. XRD patterns performed on (a) uniaxially cold pressed LPSCl pellets at 380 MPa, and on post sintered LPSCl pellets at 380 MPa, 5.10^-1^ mbar for 6 hours at (b) 60 °C, (c) 80 °C, and (d) 100 °C, and the referenced XRD pattern of LPSCl, reproduced with permission.^[1]^ Copyright 2020, American Chemical Society.


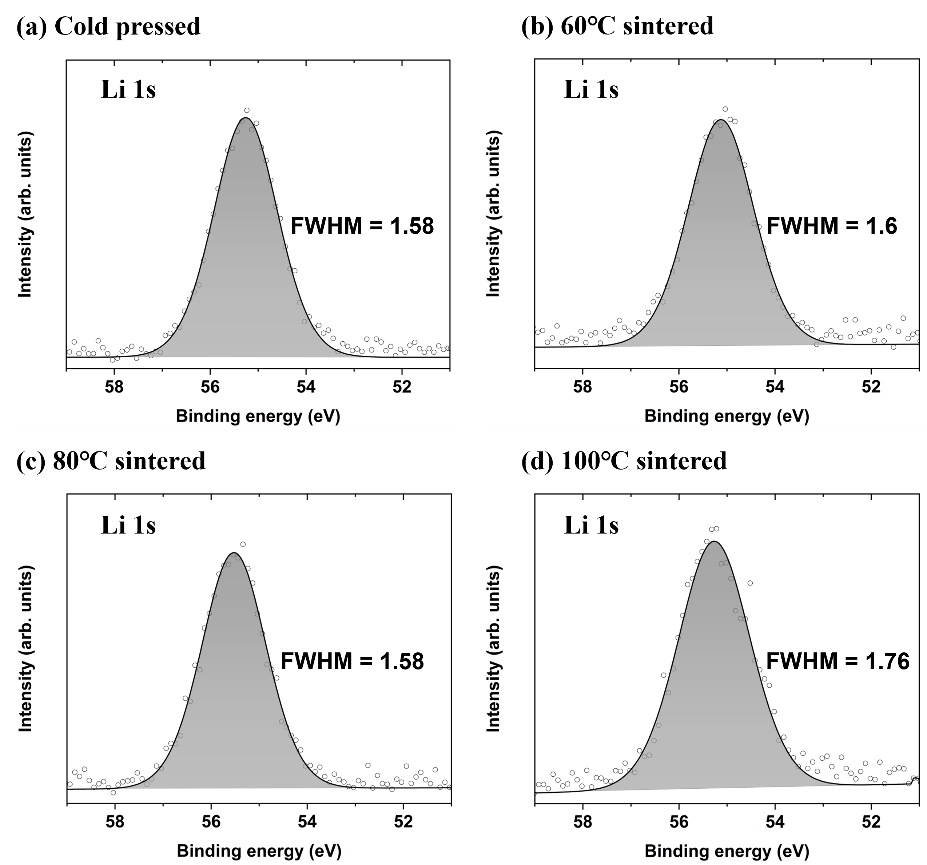


Figure_S 2. Li 1s XPS spectra acquired on (a) cold pressed LPSCl, (b) 60 °C sintered, (c) 80 °C sintered, and (d) 100 °C sintered LPSCl.


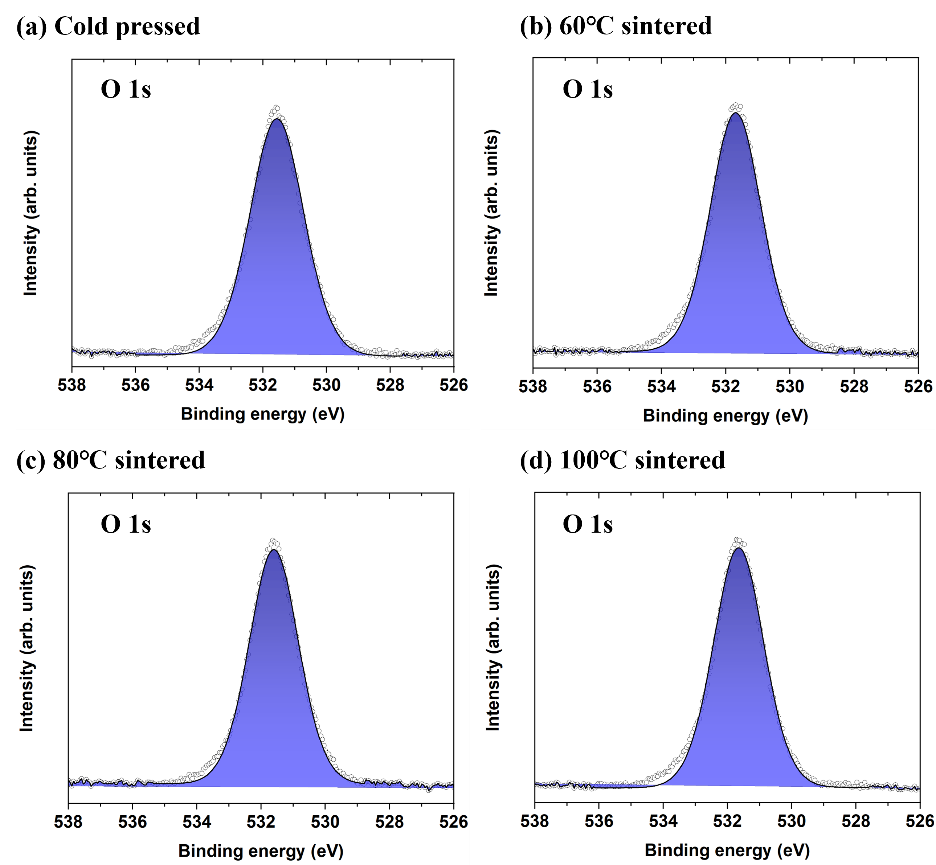


Figure_S 3. O 1s XPS spectra acquired on (a) cold pressed LPSCl, (b) 60 °C sintered, (c) 80 °C sintered, and (d) 100 °C sintered LPSCl.


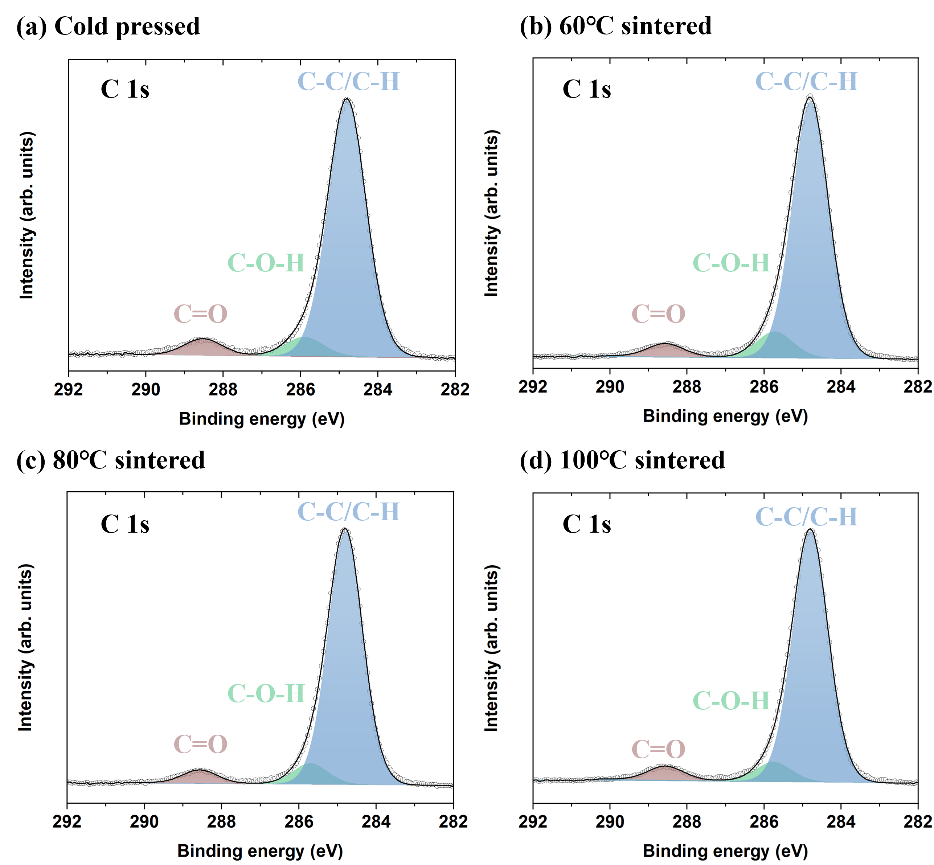


Figure_S 4. C 1s XPS spectra acquired on (a) cold pressed LPSCl, (b) 60 °C sintered, (c) 80 °C sintered, and (d) 100 °C sintered LPSCl.


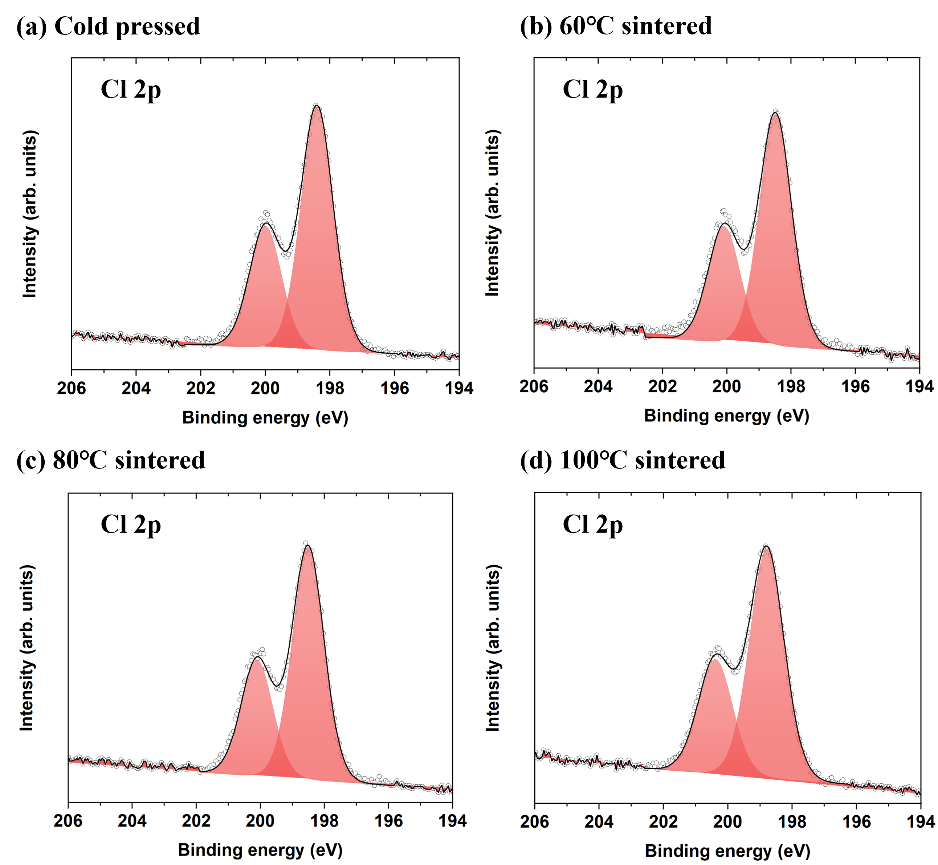


Figure_S 5. Cl 2p XPS spectra acquired on (a) cold pressed LPSCl, (b) 60 °C sintered, (c) 80 °C sintered, and (d) 100 °C sintered LPSCl.


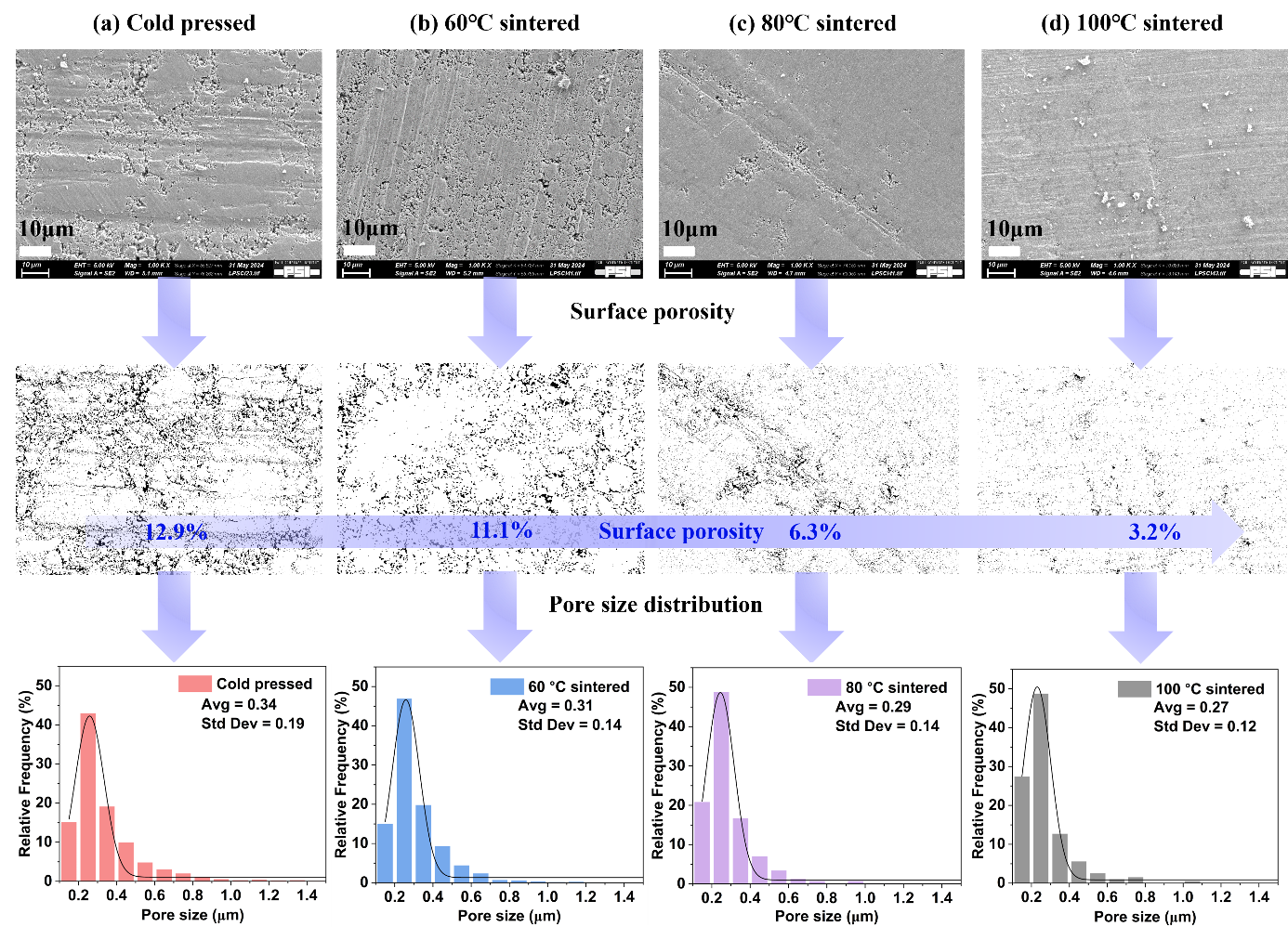


Figure_S 6. Surface morphological analysis based on top-view SEM images acquired on LPSCl pellets: (a) cold pressed, (b) 60 °C sintered, (c) 80 °C sintered, and (d) 100 °C sintered. ImageJ software was used to identify and statistically analyze surface pores, with pore size calculated based on equivalent circular areas.


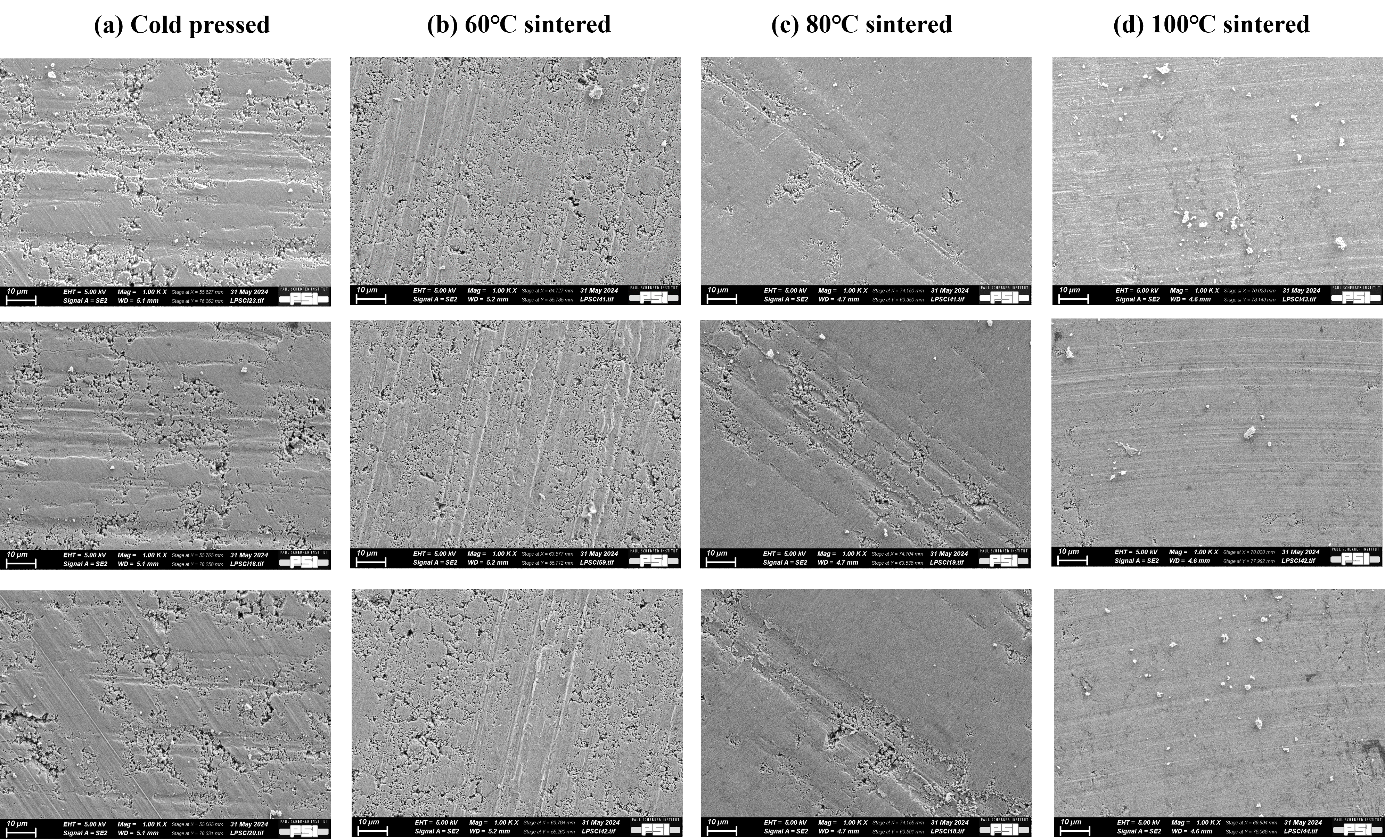


Figure_S 7. The top-view SEM images for reproducibility checks of (a) cold pressed LPSCl, (b) 60 °C sintered, (c) 80 °C sintered, and (d) 100 °C sintered LPSCl.


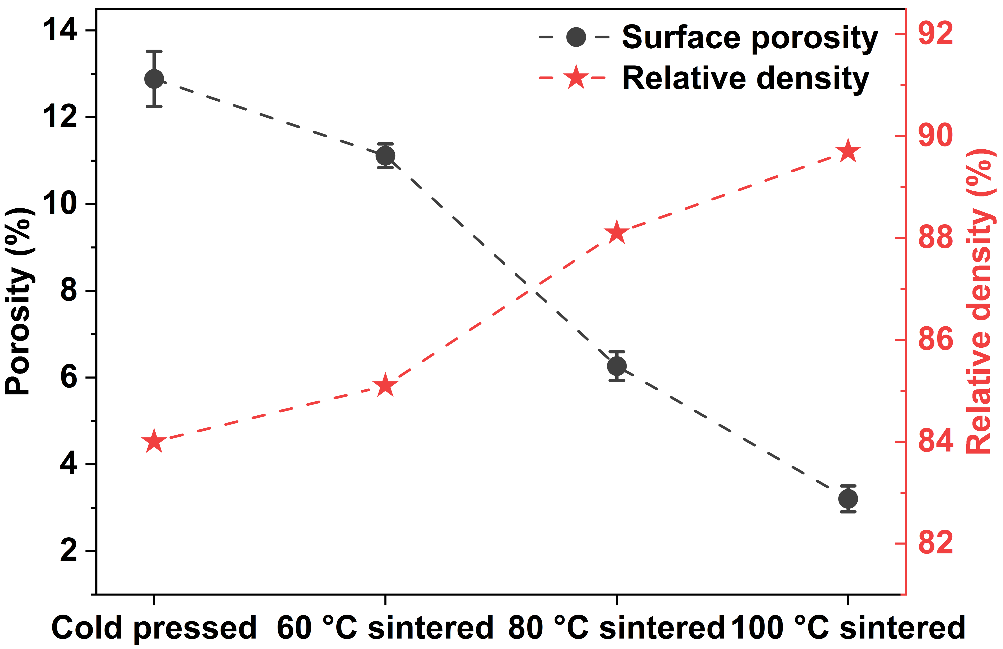


Figure_S 8. The impacts of sintering treatment on the surface porosity and bulk relative density of LPSCl pellets.


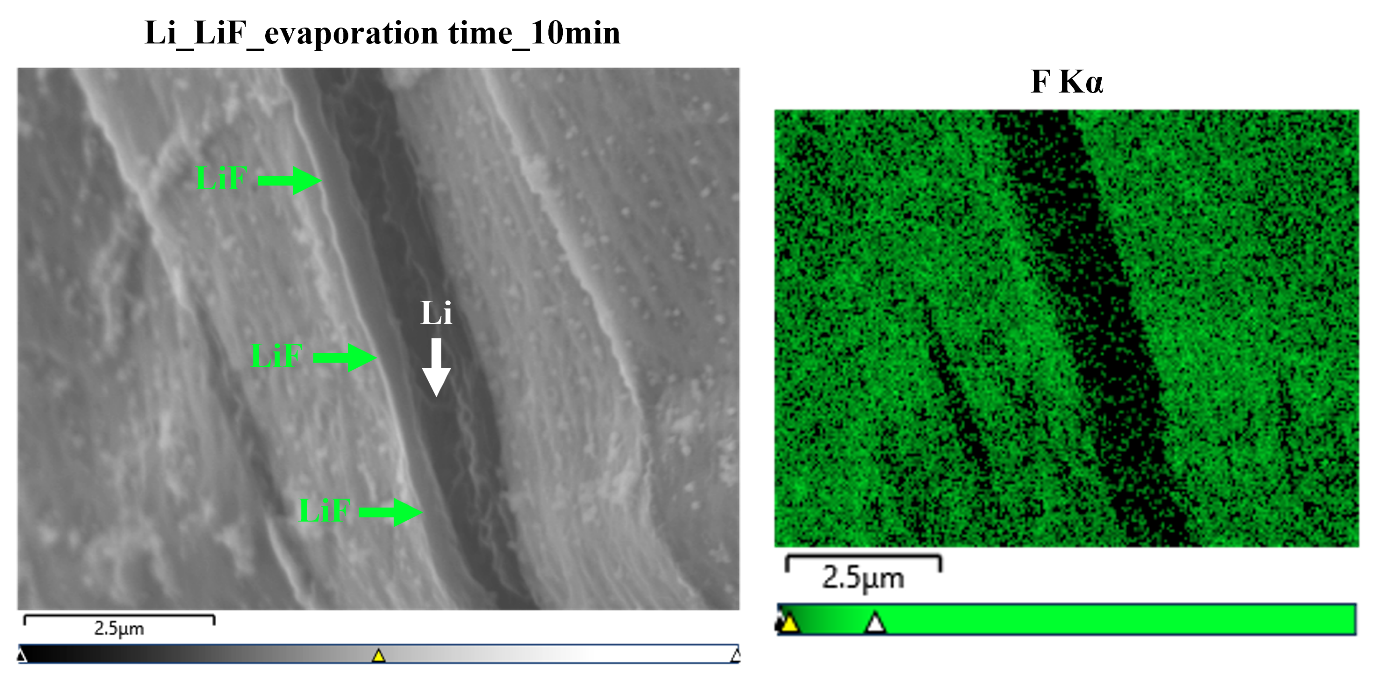


Figure_S 9. SEM image and corresponding EDX mapping of F Kα performed on lithium metal with LiF passivation layer (evaporation time of 10 minutes).

Table_S1. Statistical analysis of LiF layer thickness corresponding to different evaporation time.

| Evaporation time  (min) | Thickness (nm) | | | Slope of Fitting line |
| --- | --- | --- | --- | --- |
|  | Measured on 3 spots | Mean value | Standard deviation |  |
| 6 | 37.3, 46.5, 47.4 | 43.7 | 5.6 | 6.5$\pm$0.1 |
| 10 | 62.5, 67.7, 70.8 | 67.0 | 4.2 |  |
| 15 | 94.9, 100.2, 102.3 | 99.1 | 3.8 |  |
| 20 | 121, 124.8, 133.2 | 126.3 | 6.2 |  |


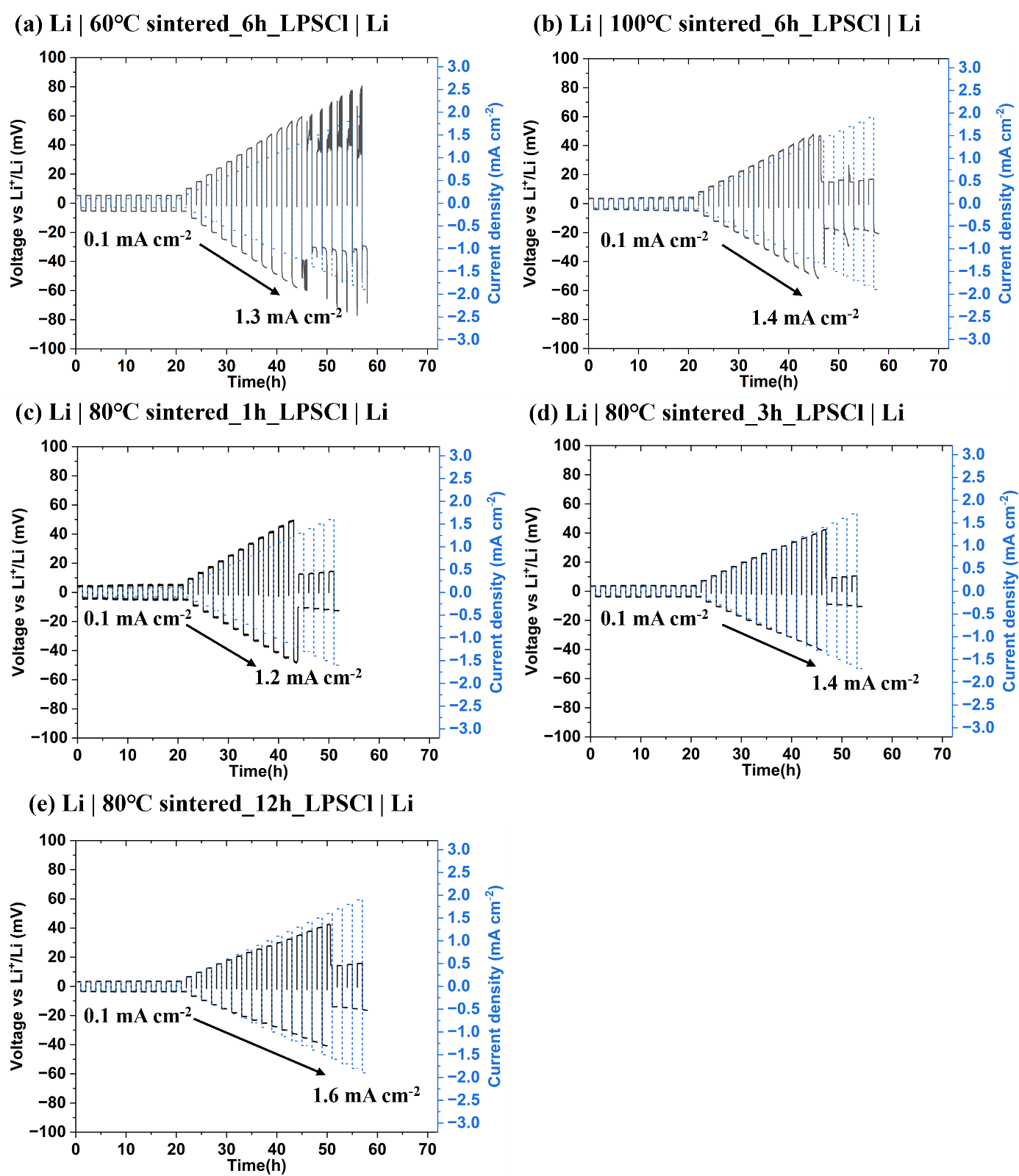


Figure_S 10. Critical current density using Li|Li symmetric cells with pristine lithium metal performed on (a) LPSCl sintered at 60 °C for 6 hours, (b) LPSCl sintered at 100 °C for 6 hours, (c) LPSCl sintered at 80 °C for 1 hour, (d) LPSCl sintered at 80 °C for 3 hours, (e) LPSCl sintered at 80 °C for 12 hours.


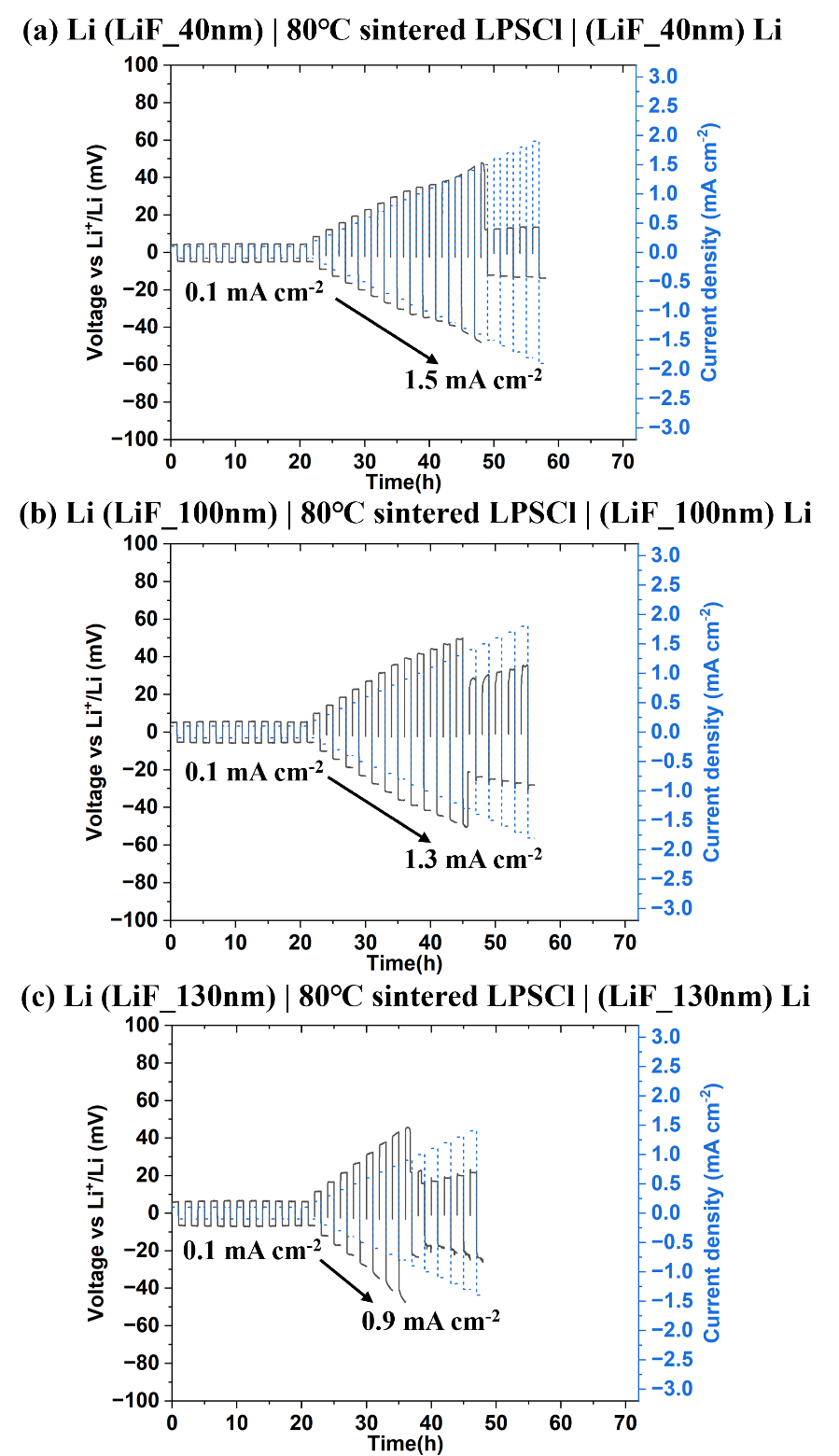


Figure_S 11. Critical current density using Li symmetric cell Li|LPSCl|Li performed on 80°C sintered LPSCl combined with various LiF -coated metallic lithium: (a) 40 nm, (b) 100 nm, (c)130 nm.


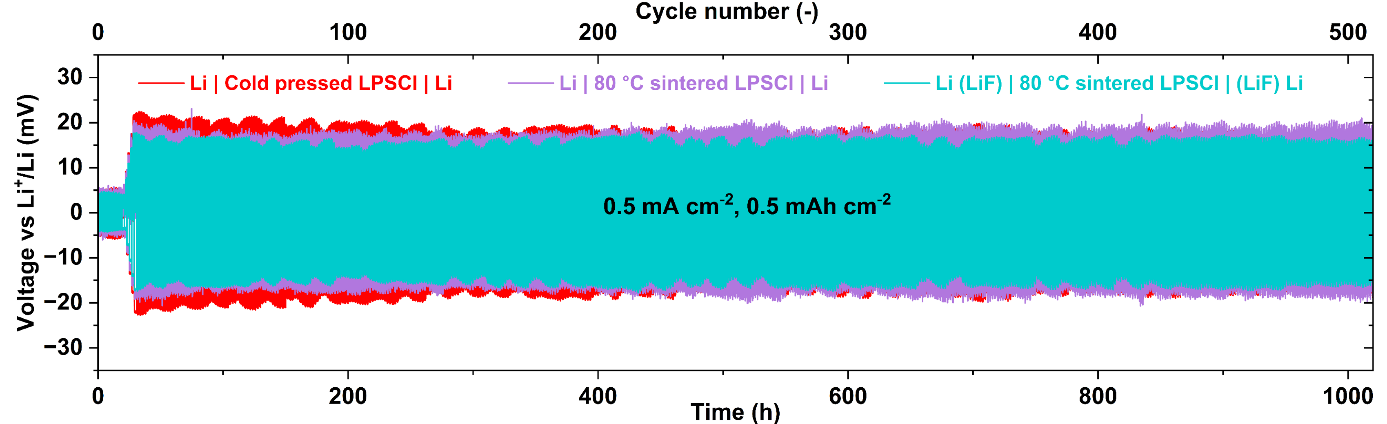


Figure_S 12. Long-term galvanostatic cycling stability at 0.5 mA cm^-2^ and 0.5 mAh cm^-2^ performed on Li symmetric cells Li|LPSCl|Li including: pristine lithium with cold pressed LPSCl (red), pristine lithium with 80 °C sintered LPSCl (purple), and lithium with LiF layer (65 nm) with 80 °C sintered LPSCl.


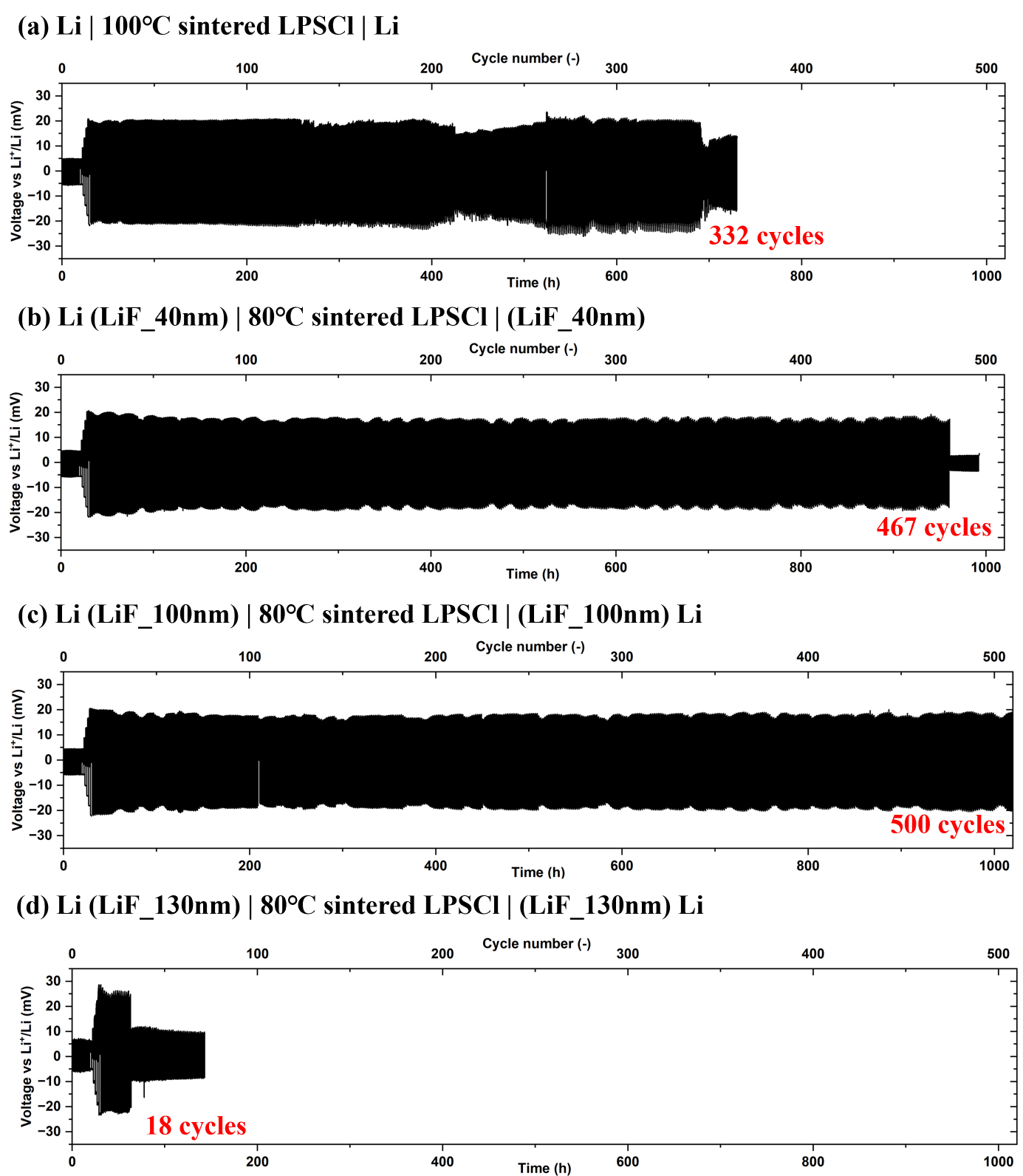


Figure_S 13. Long-term galvanostatic cycling stability at 0.5 mA cm^-2^ and 0.5 mAh cm^-2^, performed on Li symmetric cells Li|LPSCl|Li including: (a) 100 °C sintered LPSCl with pristine lithium, and 80°C sintered LPSCl with various LiF-coated lithium thickness (b) 40 nm, (c) 100 nm, (d)130 nm.


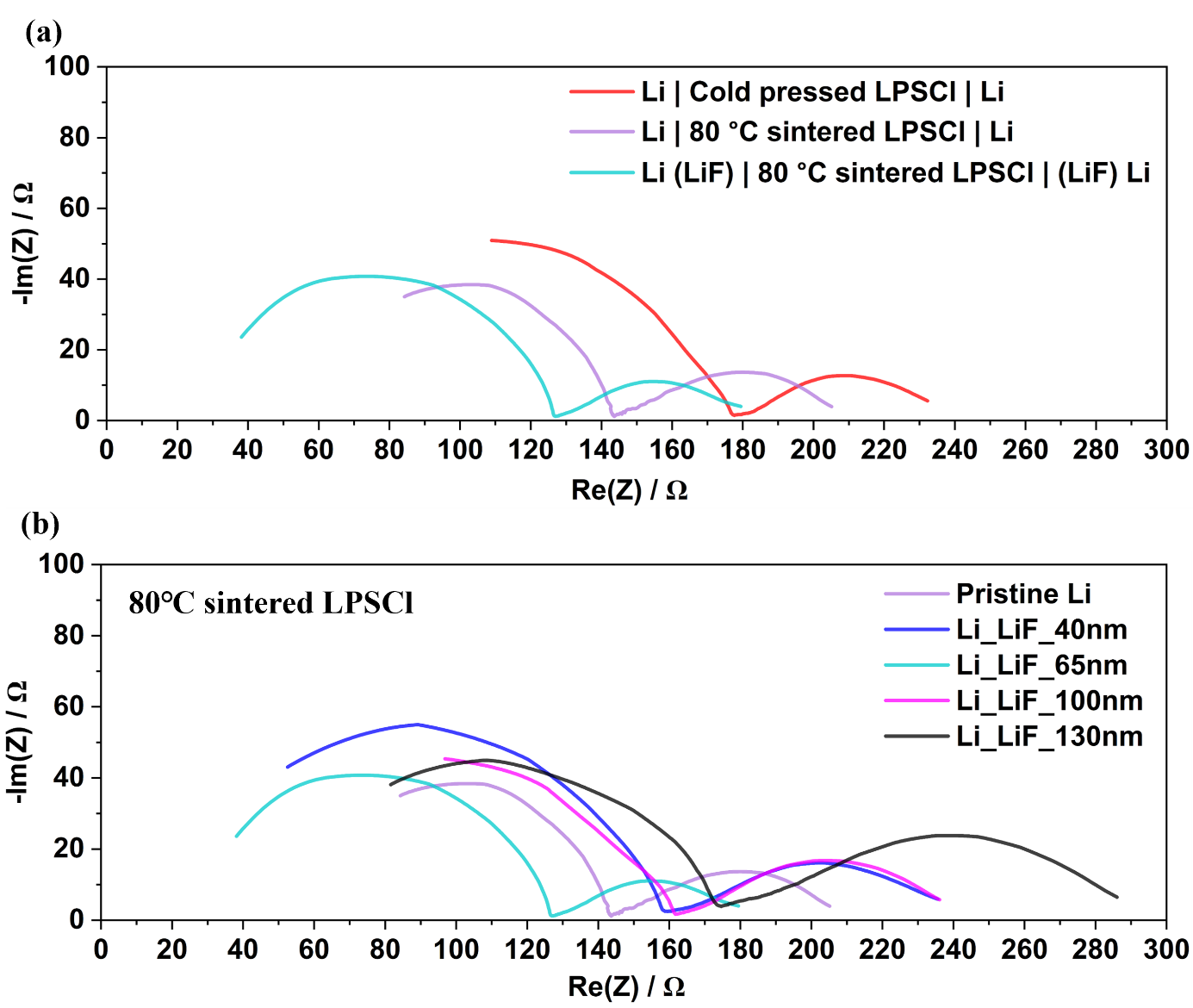


Figure_S 14. The EIS spectra comparison of lithium symmetric cells Li|LPSCl|Li before cycling for the following configurations: (a) pristine lithium with cold pressed LPSCl, pristine lithium with 80 °C sintered LPSCl, and 65 nm LiF-coated lithium with 80 °C sintered LPSCl. (b) LiF-coated lithium thickness dependency paired with 80 °C sintered LPSCl.


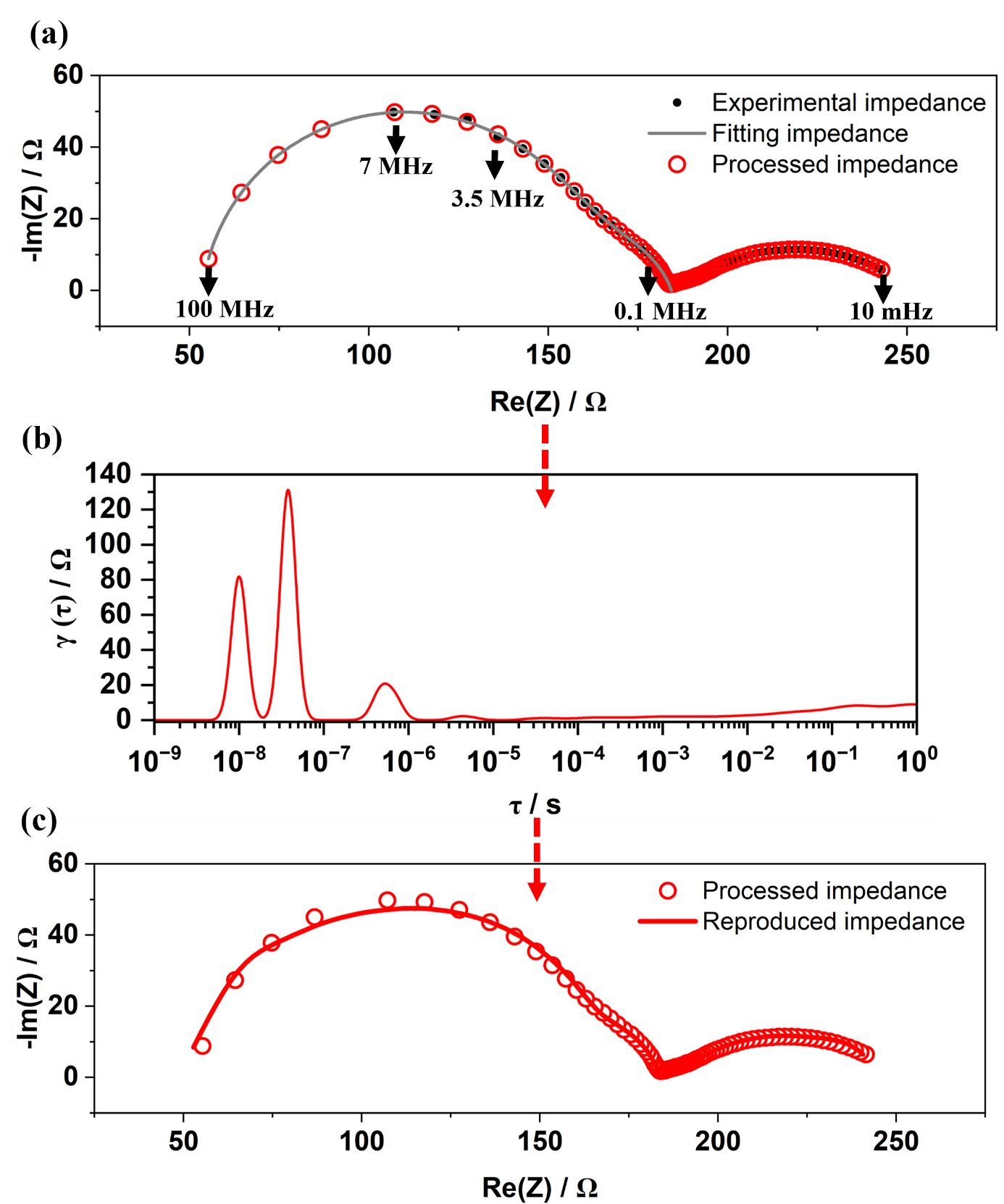


Figure_S 15. The general procedure of DRT processing. (a) Exemplary impedance spectra from Figure S14. a: Li|cold pressed LPSCl|Li, and the corresponded fitting impedance, (b) computed DRT pattern using the determined λ and μ values, (c) validation of computed DRT by reproduction of the impedance.

**Note: Procedure of DRT processing:**

The Distribution of Relaxation Times (DRT) analysis was employed to study the underlying kinetic processes by transforming the EIS data from the frequency domain to the time domain. The impedance data of the cell Li|cold pressed LPSCl|Li, as shown in **Figure S14. a**, was used as a representative example to illustrate the DRT processing procedure in this study. As depicted in **Figure S15. a**, the frequency range of experimental data was from 10 mHz to 7 MHz. The high frequency region (1 MHz ~ 100 MHz), which corresponds to the electrochemical response of the solid electrolyte, contributes significantly to the overall impedance of lithium symmetric cells.^[2]^ However, this region was only partially captured and affected by experimental noise, leading to inaccuracies in the DRT analysis.^[3]^ To better capture the kinetic processes of solid electrolyte, an equivalent circuit (EC) model was fitted to the experimental data in the range from 0.1 MHz to 7 MHz and extrapolated to 100 MHz. The fitting impedance showed excellent agreement with the experimental impedance. Subsequently, the processed impedance was constructed by combining the high frequency data (3.5 MHz to 100 MHz) from the fitting impedance with the experimental data from 10 mHz to 3.5 MHz. Based on the processed impedance, the corresponding DRT pattern was displayed in **Figure S15. b**, using a Gaussian basis function for discretization. A regularization parameter λ of 10^-5^ was applied to ensure sufficient peak separation resolution without overfitting. Additionally, a shape factor μ of 0.5 was used across all DRT pattern computations in this study. To validate the reliability of the DRT results, the reproduced impedance derived from the DRT patterns was compared with the processed impedance data, showing good agreement (**Figure S15. c**). The above-mentioned EC model fitting, and DRT computation, were performed using RelaxIS software (rhd instruments).


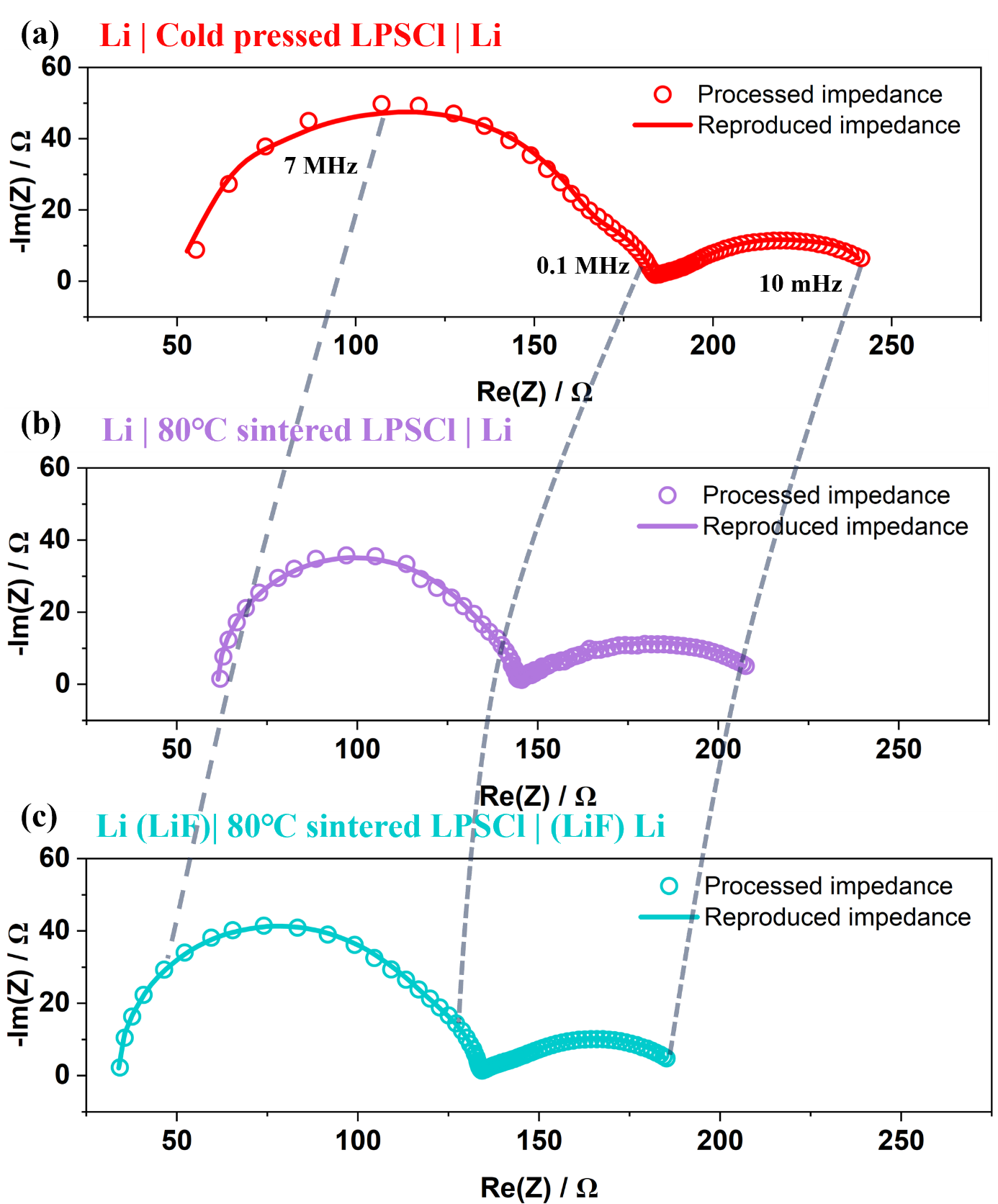


Figure_S 16. Validation of computed DRT by reproducing the impedance response for (a) pristine lithium with cold pressed LPSCl, (b) pristine lithium with 80 °C sintered LPSCl, (c) 65 nm LiF-coated lithium with 80 °C sintered LPSCl.


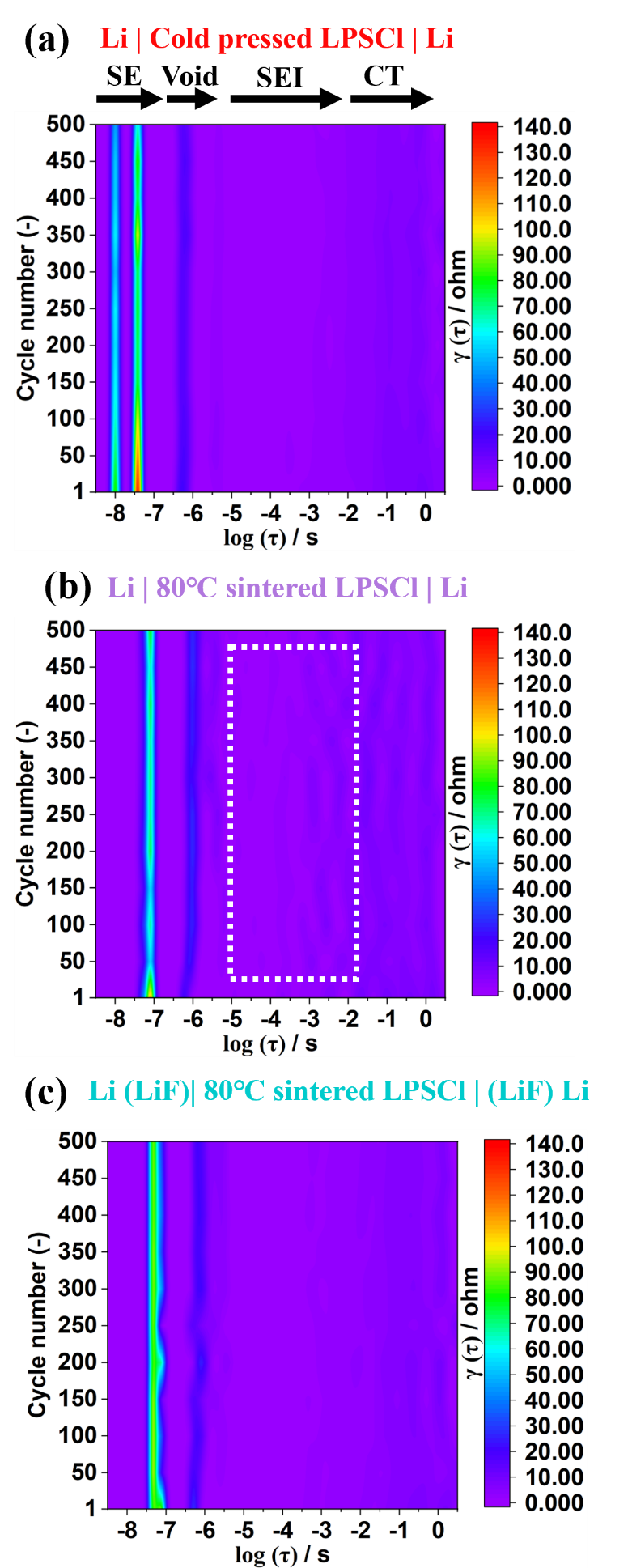


Figure_S 17. Evolution of DRT patterns depicted in contour map corresponding to Figure 5. (a), (b), (c), respectively.


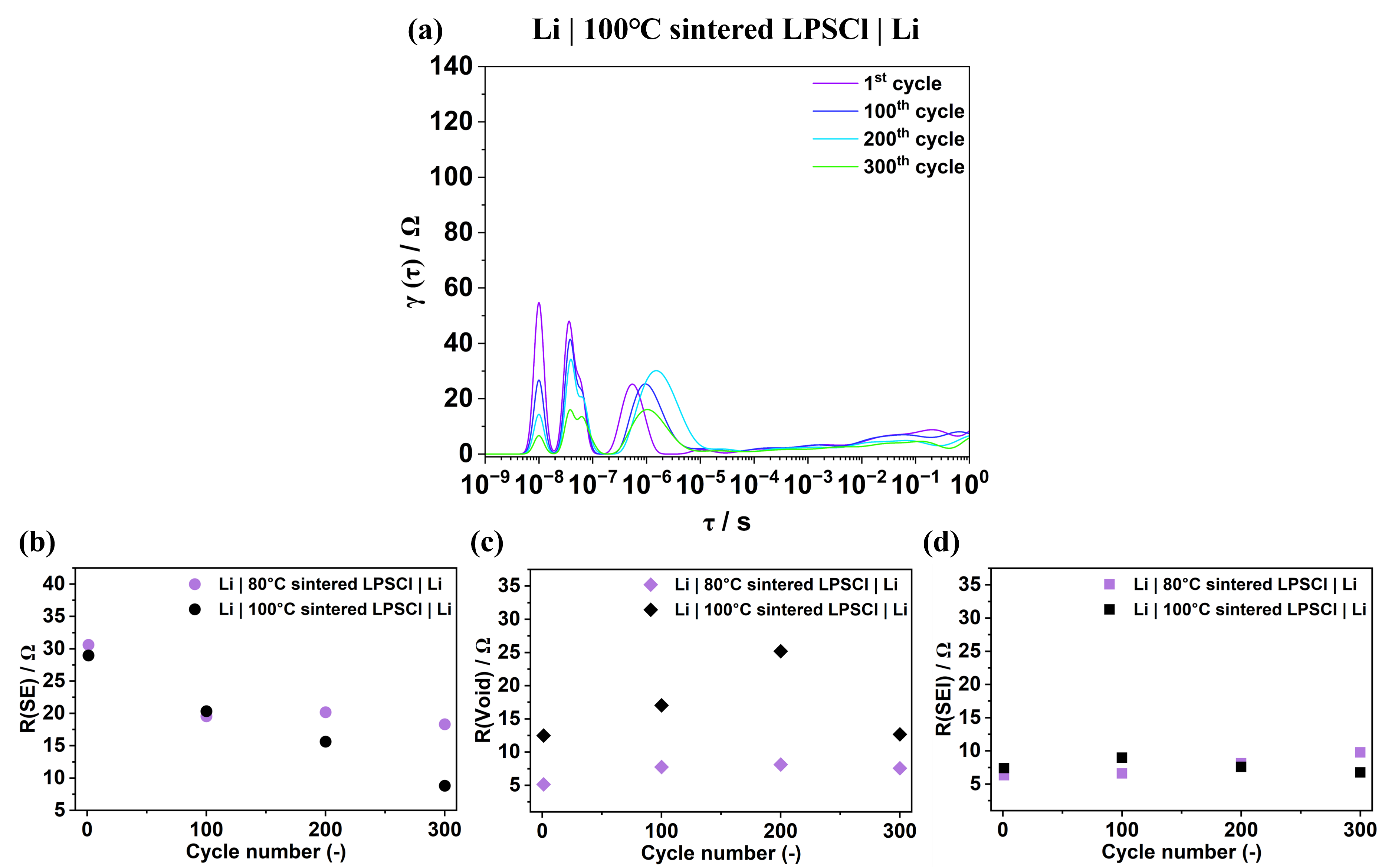


Figure_S18. DRT analysis of Li|LPSCl|Li symmetric cell employing 100 °C-sintered LPSCl during cycling at 0.5 mA cm^-2^ and 0.5 mAh cm^-2^. (a) Evolution of DRT patterns depicted in line shape. Comparison of resistances between 100 °C-sintered LPSCl and 80 °C sintered LPSCl: (b) resistance of solid electrolyte (SE), (c) resistance of void, (d) resistance of SEI.


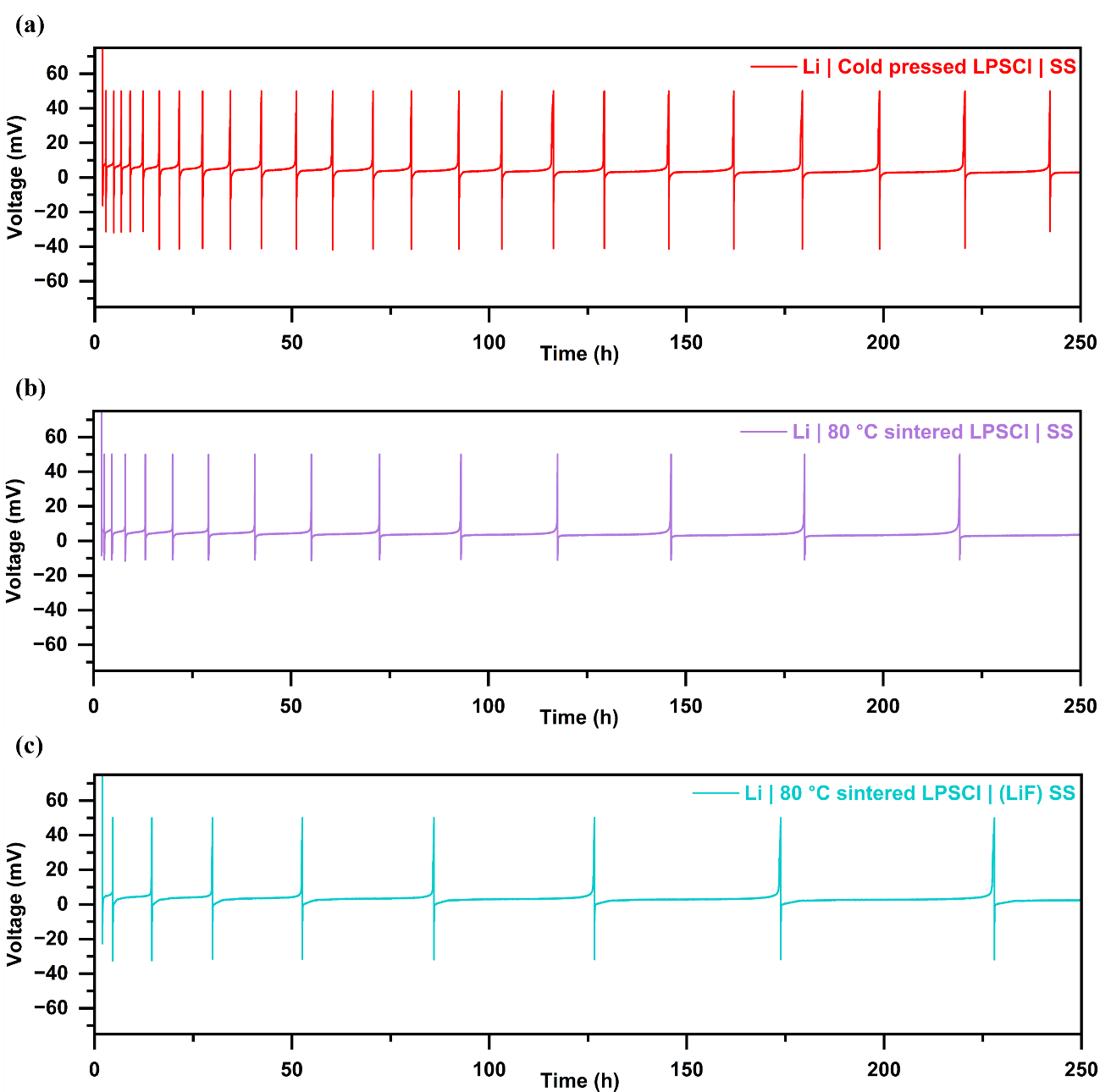


Figure_S 19. Voltage profiles of CTTA measurements performed on Li|LPSCl|stainless steel (SS). (a) pristine lithium with cold pressed LPSCl and SS, (b) pristine lithium with 80 °C sintered LPSCl and SS, (c) pristine lithium with 80 °C sintered LPSCl and 65 nm LiF-coated SS.


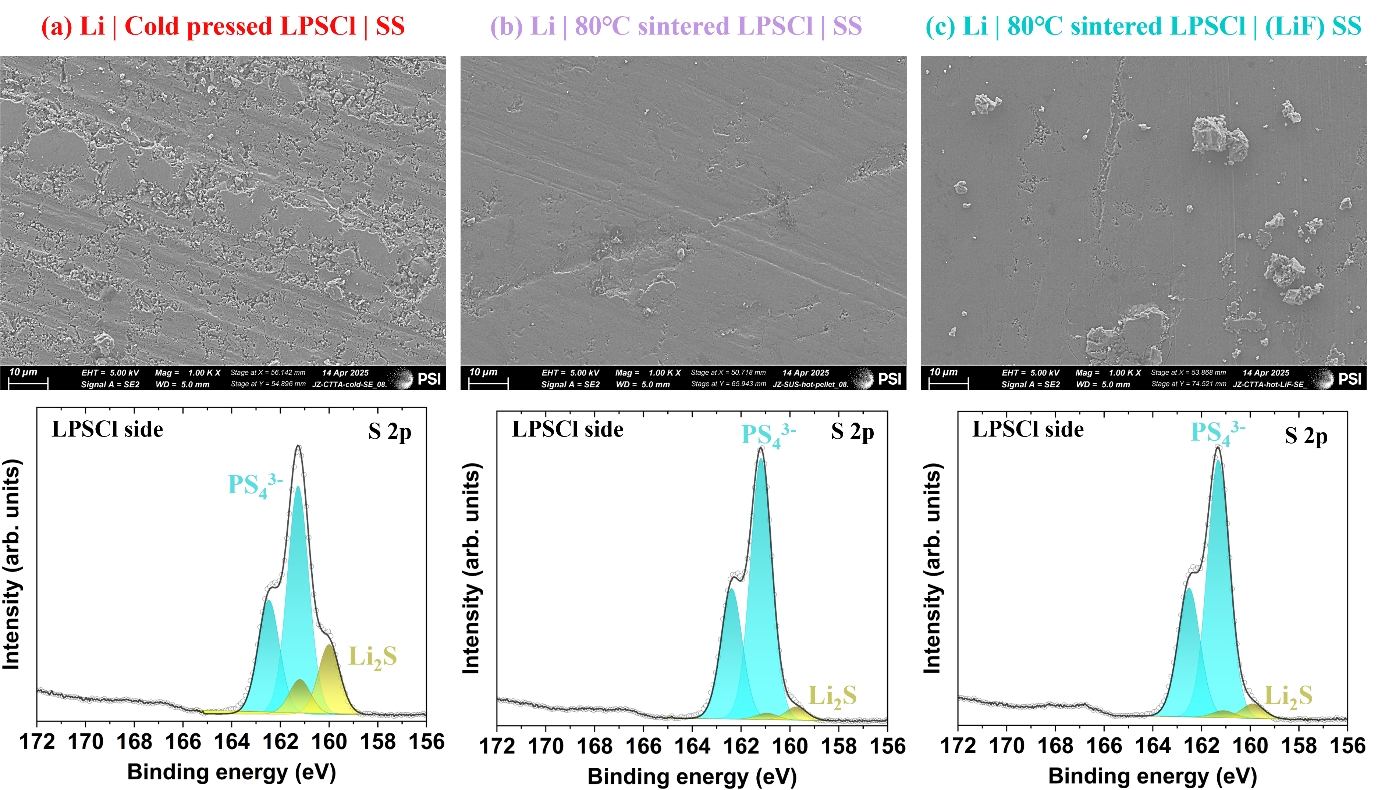


Figure_S 20. SEM images and S 2p XPS spectra acquired on LPSCl pellet sides disassembled from the Li|LPSCl|SS cells after the CTTA experiments. (a) pristine lithium with cold pressed LPSCl and SS, (b) pristine lithium with 80 °C sintered LPSCl and SS, (c) pristine lithium with 80 °C sintered LPSCl and 65 nm LiF-coated SS.


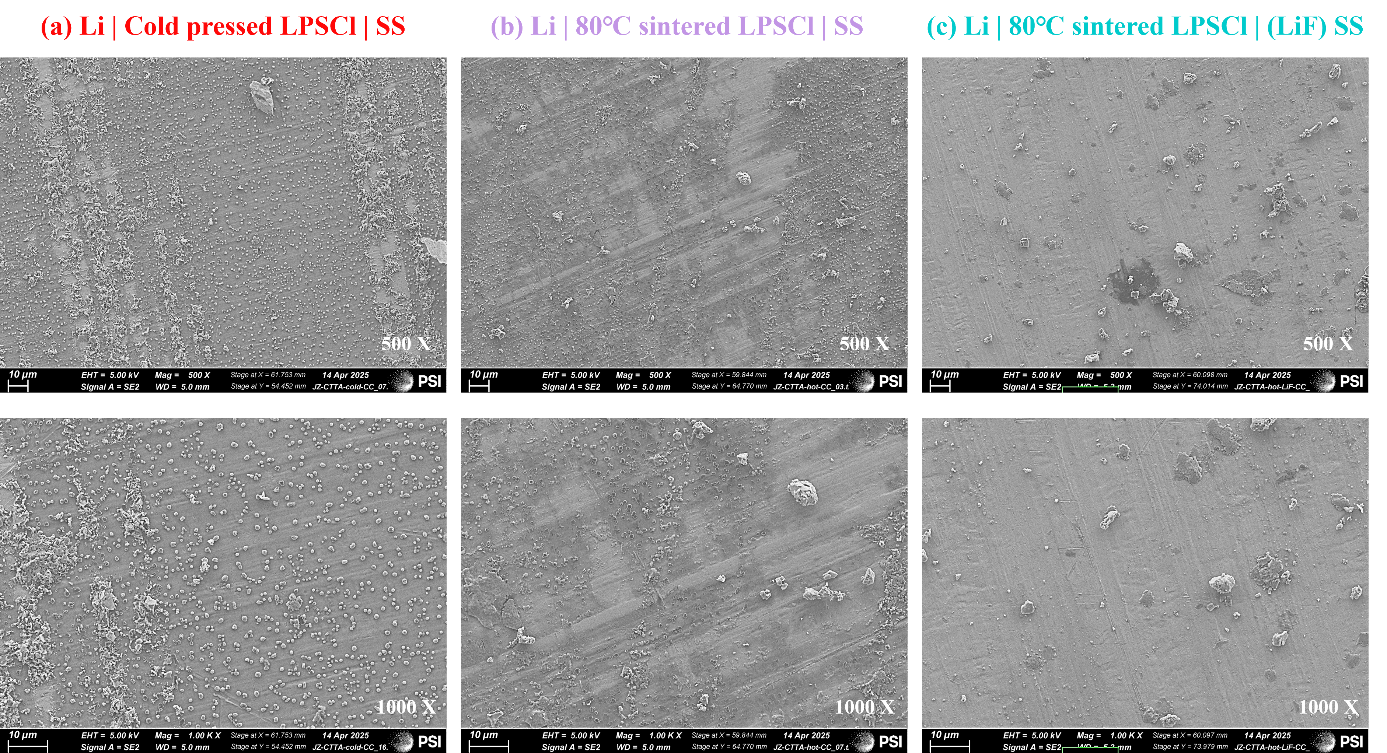


Figure_S 21. SEM images of SS sides disassembled from the Li|LPSCl|SS cells after the CTTA experiments, shown as lower magnification views (500 X and 1000 X), corresponding to Figure 6. (e), (f). (g), respectively.

.


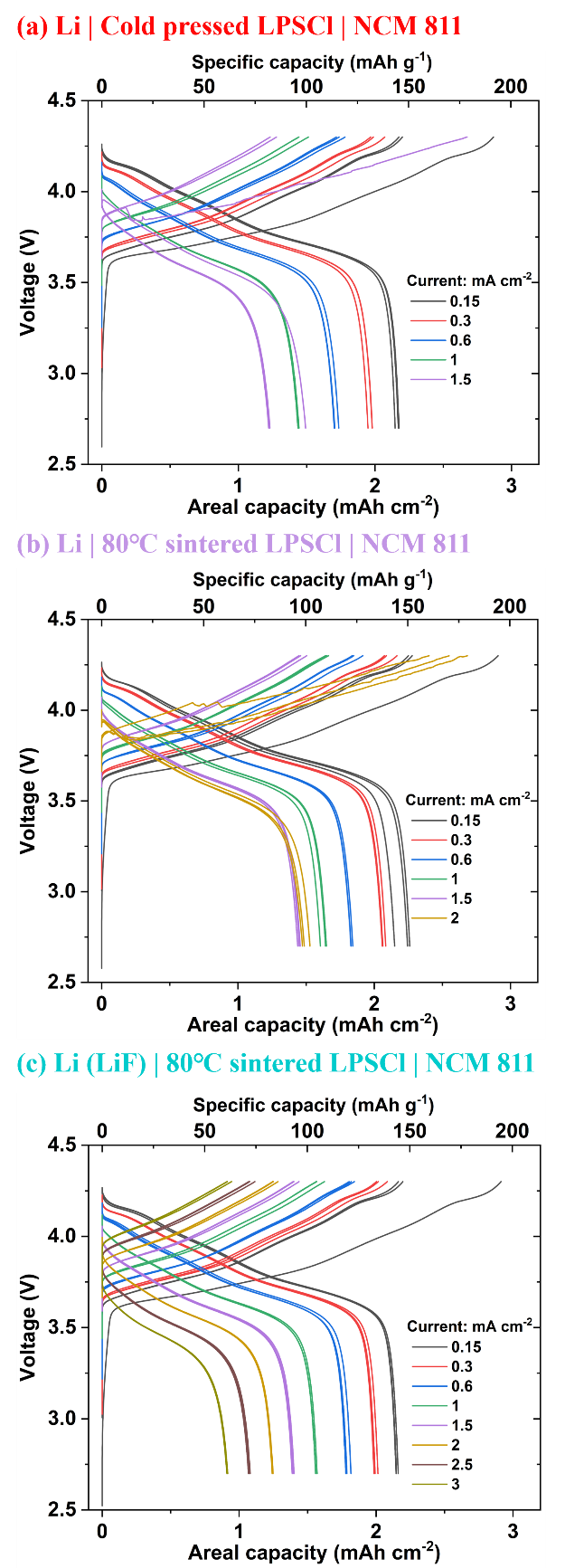


Figure_S 22. The charge-discharge voltage profiles of rate capability tests for the NCM811|LPSCl|Li full cells. (a) pristine lithium with cold pressed LPSCl, (b) pristine lithium with 80 °C sintered LPSCl, (c) 65 nm LiF-coated lithium with 80 °C sintered LPSCl.


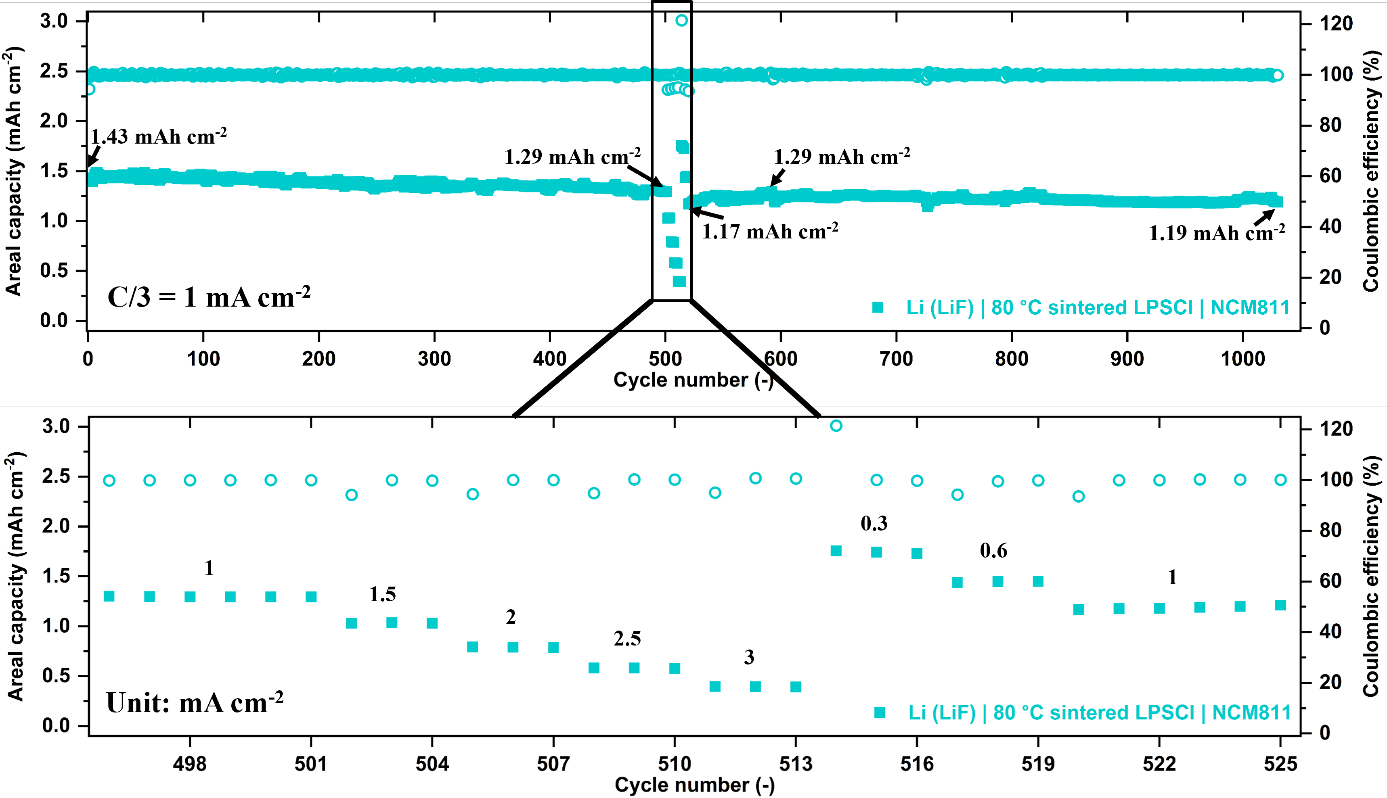


Figure_S 23. Cycling performance of (Li (LiF)|80 °C sintered LPSCl|NCM811) full cell at C/3 (1 mA cm^-2^) for 1000 cycles, with C-rate increasing procedure after 500 cycles.

Table_S 2. Comparative table summarizing recent studies using sulfide-based solid electrolytes and lithium metal anodes, highlighting the cycling performance of full cells.

| Electrolyte | Anode | Cathode | Current density mA/cm^2^ | Areal capacity mAh/cm^2^ | Cycle life | Capacity retention % | Cycle  condition | Ref. |
| --- | --- | --- | --- | --- | --- | --- | --- | --- |
| Li_6_PS_5_Cl  (MgF_2_) | Li foil | LiCoO_2_ | 0.2 | 1.2 | 100 | 93.3 | 25 ºC | [4] |
| Li_6_PS_5_Cl  (Li_3_TCA) | Li foil | LiNi_0.8_Co_0.1_Mn_0.1_O_2_ (LiNbO_3_ coating) | 0.9 | 1.65 | 500 | 47 | 20 MPa, RT | [5] |
| Li_6_PS_5_Cl  (Sintered @ 550°C) | Li foil | LiCoO_2_  (LiNbO_3_ Coating) | 0.35 | 0.31 | 100 | 80.3 | 25 ºC | [6] |
| Li_6_PS_5_Cl | Li foil  (CuF_2_-PVDF-HFP) | LiCoO_2_  (Li_2_ZrO_3_ coating) | 1 | 0.83 | 300 | 82.19 | 55 ºC | [7] |
| Li_6_PS_5_Cl | Li foil  (MgF_2_) | LiNi_0.7_Co_0.15_Mn_0.15_O_2_ | 0.78 | 1.7 | 800 | 82 | 15 MPa  30 ºC | [8] |
| Li_5.5_PS_4.5_Cl_1.5_  (LiTFSI1) | Li foil | LiCoO_2_ | 0.6 | 0.2 | 1000 | 90.2 | 30 ºC | [9] |
| Li_6_PS_5_Cl  (PEGDME) | Li foil | LiCoO_2_  (LiZrOx coating) | 0.5 | 0.39 | 2600 | 58 | - | [10] |
| Li_5.5_PS_4.5_Cl_1.5_  (Li_3_N) | Li foil | LiNi_0.8_Co_0.1_Mn_0.1_O_2_ | 0.3 | 0.88 | 260 | 80 | 5 MPa 50ºC | [11] |
| Li_6_PS_5_Cl | Li foil  (AgNO_3_-FEC) | LiNi_0.6_Co_0.2_Mn_0.2_O_2_ (Li_3_B_11_O_18_ coating) | 1.07 | 1.41 | 300 | 82.7 | 5 MPa,  RT | [12] |
| Li_6_PS_5_Cl (Li_9.54_[Si_0.5_Sn_0.5_]PSBrO) | Li foil | LiCoO_2_ | 0.5 | 0.93 | 500 | 95.04 | 30 MPa  55 ºC | [13] |
| Li_6_PS_5_Cl  (Sintered @ 80°C) | Li foil  (LiF) | LiNi_0.8_Co_0.1_Mn_0.1_O_2_ | 1 | 1.46 | 2600 | 64 | 20 MPa  RT | This work |
| Li_6_PS_5_Cl  (Sintered @ 80°C) | Li foil  (LiF) | LiNi_0.8_Co_0.1_Mn_0.1_O_2_ | 1.5 | 1.36 | 450 | 91 | 20 MPa  RT | This work |

**References**

1. Minafra, N., M.A. Kraft, T. Bernges, C. Li, R. Schlem, B.J. Morgan, and W.G. Zeier, *Local Charge Inhomogeneity and Lithium Distribution in the Superionic Argyrodites Li6PS5X (X = Cl, Br, I).* Inorganic Chemistry, 2020. **59**(15): p. 11009-11019.

2. Chen, H., H. Zhang, Y. Zhou, J. Chen, X. Huang, and B. Tian, *Structure-conduction correlations in a chlorine-rich superionic lithium-argyrodite solid electrolyte: A DRT analysis.* Journal of Power Sources, 2023. **583**: p. 233579.

3. Tang, Z., A. Morchhale, J.R. Sayre, and J.-H. Kim, *In-situ electrochemical characterization of dynamic void formation and lithium dendrite in all-solid-state batteries.* Journal of Energy Storage, 2025. **107**: p. 114966.

4. Liu, C., B. Chen, T. Zhang, J. Zhang, R. Wang, J. Zheng, Q. Mao, and X. Liu, *Electron Redistribution Enables Redox-Resistible Li6PS5Cl towards High-Performance All-Solid-State Lithium Batteries.* Angewandte Chemie International Edition, 2023. **62**(22): p. e202302655.

5. Braks, L., J. Zhang, A. Forster, P. Fritz, J. Oh, M. El Kazzi, J.W. Choi, and A. Coskun, *Interfacial Stabilization by Prelithiated Trithiocyanuric Acid as an Organic Additive in Sulfide-Based All-Solid-State Lithium Metal Batteries.* Angewandte Chemie International Edition, 2024. **63**(35): p. e202408238.

6. Liu, G., W. Weng, Z. Zhang, L. Wu, J. Yang, and X. Yao, *Densified Li6PS5Cl Nanorods with High Ionic Conductivity and Improved Critical Current Density for All-Solid-State Lithium Batteries.* Nano Letters, 2020. **20**(9): p. 6660-6665.

7. Gao, Q., D. Wu, X. Zhu, P. Lu, T. Ma, M. Yang, L. Chen, H. Li, and F. Wu, *Dendrite-free lithium-metal all-solid-state batteries by solid-phase passivation.* Nano Energy, 2023. **117**: p. 108922.

8. Lim, H., S. Jun, Y.B. Song, K.H. Baeck, H. Bae, G. Lee, J. Kim, and Y.S. Jung, *Rationally Designed Conversion-Type Lithium Metal Protective Layer for All-Solid-State Lithium Metal Batteries.* Advanced Energy Materials, 2024. **14**(12): p. 2303762.

9. Zhang, S., Q. Li, J. Gao, R. Liu, X. Jiang, S. Xiong, C. Wang, Z. Zhang, Y. Qiu, Y. Shi, L. Yin, and R. Wang, *Melt-Infusion-Induced Electrolyte Surface Coating Stabilized Sulfide-Based All-Solid-State Lithium Metal Batteries.* ACS Nano, 2025. **19**(11): p. 10912-10921.

10. Yang, X., X. Gao, M. Jiang, J. Luo, J. Yan, J. Fu, H. Duan, S. Zhao, Y. Tang, R. Yang, R. Li, J. Wang, H. Huang, C. Veer Singh, and X. Sun, *Grain Boundary Electronic Insulation for High-Performance All-Solid-State Lithium Batteries.* Angewandte Chemie International Edition, 2023. **62**(5): p. e202215680.

11. Ren, P., X. Wang, B. Huang, Z. Liu, and R. Liu, *Li3N interlayer enables stable long-term cycling for sulfide-based all-solid-state Li metal batteries.* Journal of Energy Storage, 2024. **82**: p. 110200.

12. Liang, Y., C. Shen, H. Liu, C. Wang, D. Li, X. Zhao, and L.-Z. Fan, *Tailoring Conversion-Reaction-Induced Alloy Interlayer for Dendrite-Free Sulfide-Based All-Solid-State Lithium-Metal Battery.* Advanced Science, 2023. **10**(19): p. 2300985.

13. Xu, F., Y. Wu, L. Wang, Z. Zhang, G. Liu, C. Guo, D. Wu, C. Yi, J. Luo, W. He, C. Xu, M. Yang, H. Li, L. Chen, and F. Wu, *Low-Pressure Sulfide All-Solid-State Lithium-Metal Pouch Cell by Self-Limiting Electrolyte Design.* Advanced Energy Materials, 2025. **15**(23): p. 2405369.
